# A unified framework for species spatial patterns: Linking the occupancy area curve, Taylor's Law, the neighborhood density function and two‐plot species turnover

**DOI:** 10.1111/ele.13788

**Published:** 2021-08-04

**Authors:** Justin Kitzes, Micah Brush, Kyle Walters

**Affiliations:** ^1^ Department of Biological Sciences University of Pittsburgh Pittsburgh PA USA; ^2^ Department of Physics University of California Berkeley Berkeley CA USA

**Keywords:** commonality, distance decay, macroecology, pair correlation, point pattern, point process, Ripley's K, theory

## Abstract

The description of spatial patterns in species distributions is central to research throughout ecology. In this manuscript, we demonstrate that five of the most widely used species‐level spatial patterns are not only related, but can in fact be quantitatively derived from each other under minimal assumptions: the occupancy area curve, Taylor's Law, the neighborhood density function, a two‐plot variant of Taylor's Law and two‐plot single‐species turnover. We present an overarching mathematical framework and derivations for several theoretical example cases, along with a simulation study and empirical analysis that applies the framework to data from the Barro Colorado Island tropical forest plot. We discuss how knowledge of this mathematical relationship can support the testing of ecological theory, suggest efficient field sampling schemes, highlight the relative importance of plot area and abundance in driving turnover patterns and lay the groundwork for future unified theories of community‐level spatial metrics and multi‐patch spatial patterns.

## Graphical abstract

We show that several widely used single species spatial metrics (the occupancy area curve, Taylor's Law, the neighborhood density function and plot‐based turnover) are all derivable from each other under minimal assumptions. We provide example equations showing this relationship and demonstrate its application to simulated and empirical data.

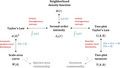

## INTRODUCTION

Describing the spatial patterns formed by individuals in a population is one of the most important and widespread research activities across ecology. Knowledge of species‐level spatial patterns is used to draw inferences about the possible underlying ecological mechanisms operating in populations and communities, design sampling schemes for characterising populations and identify general laws that are common across ecological systems (Brown, 1995; Diggle, 2013; Lawton, 1999; Magurran, 2011; Wiegand & Moloney, 2013). A diverse set of quantitative descriptions of intraspecies spatial patterns, here referred to as spatial metrics, have been developed and are in widespread use.

At the most general level, such spatial metrics can be divided into three broad categories. The first category includes metrics that describe plot‐based spatial scaling, which is the change in some property of a population with the area of a plot being surveyed. Perhaps, the most important of these scaling metrics is the occupancy area curve (He & Condit, 2007), which describes how the probability that a plot is occupied changes with the area of that plot (see also the related scale‐area curve, Kunin 1998). The community‐level species–area relationship is frequently constructed or predicted as the sum of these occupancy area curves across all species in the community (e.g. Coleman, 1981; Green & Plotkin, 2007; Harte, 2011; He & Legendre, 2002; Kitzes & Harte, 2014; Sizling & Storch, 2004). Shifting from occupancy to abundance, Taylor's Law is a well‐established relationship between the mean and variance in the abundance of a species in a plot across plot areas (Taylor, 1961). Both temporal and spatial forms of Taylor's Law have been studied, with the relationship between variance and mean often found to resemble a power law with relatively universal parameters across communities (Xiao et al., 2015).

The second broad category of spatial metrics is those that describe plot‐based spatial turnover, which is the relationship in some property of a population or community between two plots separated by a known distance. For a single species, for example the joint probability that two plots are both occupied by a species can be defined as a two‐plot analogue to the occupancy area curve. A single‐species turnover metric can then be defined as this probability of joint occupancy divided by the probability that one of the two plots is occupied. Similar to the manner in which occupancy area curves for all species sum to the species–area relationship, the sum of this single‐species two‐plot turnover metric across all species gives the Sorensen index of commonality or distance decay for that community (Soininen et al., 2007). Expanding again from occupancy to abundance, a two‐plot variant of Taylor's Law for a species can be defined as the covariance in abundance between pairs of plots as a function of the plot area and the distance between plots.

Finally, the third broad category includes spatial metrics that are not based on plots but are instead measured in continuous space. Unlike the previous two categories, which are most closely associated with spatial macroecology, these metrics originate largely with the field of point pattern analysis (Diggle, 2013; Illian et al., 2008; Wiegand & Moloney, 2013). In ecology, perhaps the most frequently used single‐species metrics of this type are Ripley's K, the O‐ring or neighborhood density function and the pair‐correlation function (Wiegand & Moloney, 2004). Each of these related metrics reflects, in some manner, the probability that two discs of infinitesimally small area separated by some distance both contain a point, representing an individual of the species. A related metric in continuous space that mixes the single species and community perspectives is the probability that two individuals separated by a known distance belong to the same species, a relationship that has been derived for communities with a variety of dispersal kernels (Chave & Leigh, 2002).

**FIGURE 1:**
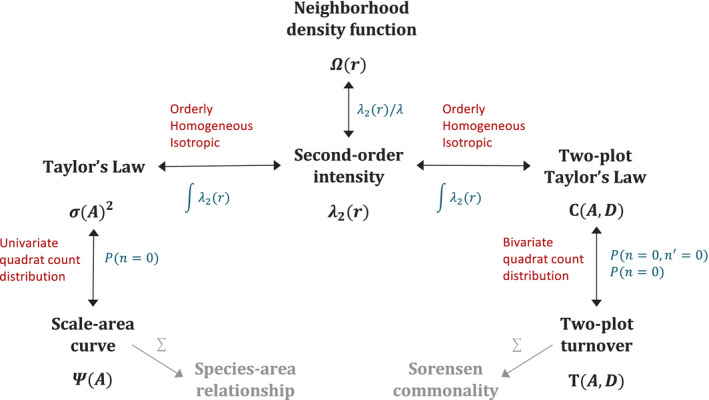
Relationships between the five focal spatial metrics and the second‐order intensity function. Arrows indicate mathematical relationships between specific metrics, which are indicated in schematic form in blue text. Red text summarizes the assumptions necessary for each mathematical relationship to hold. Grey arrows and text show the relationship between the focal single‐species metrics and two community‐level metrics, the species–area relationship and Sorensen commonality or distance decay (see *Discussion*)

The diverse spatial metrics described above are generally considered separately when describing species‐level spatial patterns, and it is often presumed that the shapes of metrics in the three categories above represent distinct aspects of a species’ spatial distribution. In this manuscript, we demonstrate that not only are all three categories of metrics mathematically related, but that with relatively minimal assumptions, knowledge of the shape of any one metric described above provides sufficient information to quantitatively derive the form of several other metrics. Below, we focus specifically on demonstrating the mathematical relationships between five of these spatial metrics for a single species: the occupancy area curve, Taylor's Law, the neighborhood density function, the two‐plot variant of Taylor's Law and two‐plot turnover (Figure 1).

This analysis builds on prior attempts to uncover relationships between these and other similar spatial metrics. For example the relationship between the pair correlation function and the variance of abundance in a plot of fixed area, or covariance in abundance between two plots of a fixed area, has appeared in several references (Diggle, 2013; Illian et al., 2008). Azaele et al., (2015) explored the relationship between the pair correlation function and the spatial scaling of plot variance in the context of exploring species–area relationships and the spatial scaling of species‐abundance distributions. Focusing more specifically on only single‐plot metrics, He and Gaston (2003) describe the relationship between Taylor's Law and the occupancy area curve. This relationship is also implicitly used in constructing the sampled species–area relationship from a species abundance distribution (Green & Plotkin, 2007; He & Legendre, 2002; Kitzes & Harte, 2014).

At the community level, several efforts have been made to link the species–area relationship to overlap in species composition between two plots, the community‐level equivalent to relating the occupancy area curve to two‐plot single species turnover across scales (Harte & Kinzig, 1997; Hui, 2009; McGlinn & Hurlbert, 2012; McGlinn et al., 2015; Tjørve & Tjørve, 2008). These efforts, however, have only succeeded in deriving this relationship for pairs of plots that share a common side or that touch at a corner, with any further relationships requiring assumptions of independence between plot abundances at certain areas or distances to complete the analysis.

We begin below by outlining the general mathematical framework that unites the five core spatial metrics named above. To demonstrate the framework, we derive analytical expressions and approximations for several theoretical examples in which knowledge of one spatial metric is used to derive the form of the remaining four. We then present the results of both simulation and empirical analyses showing the ability of the framework to link the shapes of the five spatial metrics in practice. We then discuss ways in which this newly developed framework supports more robust testing of ecological theory, suggests different and potentially more efficient field sampling schemes and demonstrates the importance of plot area and species abundance in driving measures of turnover. We conclude with several future directions for expanding this framework to the community level and to multi‐patch spatial patterns.

## METHODS AND RESULTS

### General framework

Consider a large landscape containing many individuals of a single species. The location of each individual is represented by a point, and this set of points forms a point pattern that is defined within the landscape. We assume that the point pattern is (1) orderly, such that no two points can occur at the same location, (2) homogeneous, such that the statistical properties of the pattern, particularly the first‐order and second‐order intensity (defined below), are the same across the entire landscape and (3) isotropic, such that there is no directionality in the pattern. Together, these three common assumptions define a “well‐behaved” point pattern. One or more plots or quadrats of area A that are small relative to the landscape area may be located within this landscape.

In all equations below, we use the notation F(x), where F may be a Greek or upper case Roman letter, to denote a metric or intermediate variable whose shape is a function of one or more variables x. We reserve the notation P(X=Y) specifically for the probability that a random variable X takes the value Y.

There are many methods of describing point patterns quantitatively (Illian et al., 2008; Wiegand & Moloney, 2013). Three intermediate statistics will be central to the framework presented here (see Supporting Information 1 for formal definitions). The first intermediate statistic is the pattern's first‐order intensity, λ, which describes the overall density of points in the landscape and can be calculated as the number of points in the entire pattern divided by the area of the landscape. The second intermediate statistic is the second‐order intensity, $\lambda_{2}\left(r\right)$, which represents the probability that two locations separated by distance r both contain a point. The third intermediate statistic is the quadrat count distribution (Diggle, 2013) or species‐level spatial abundance distribution (Harte, 2011), P(n|A), which describes the probability of observing n individuals in a plot of area A that is small relative to the landscape. A two‐plot joint quadrat count distribution for the simultaneous abundance in two plots, each of area A, can be defined as *P (n, n′|A,D)*, where D is the distance between the nearest edges of the two plots.

**FIGURE 2:**
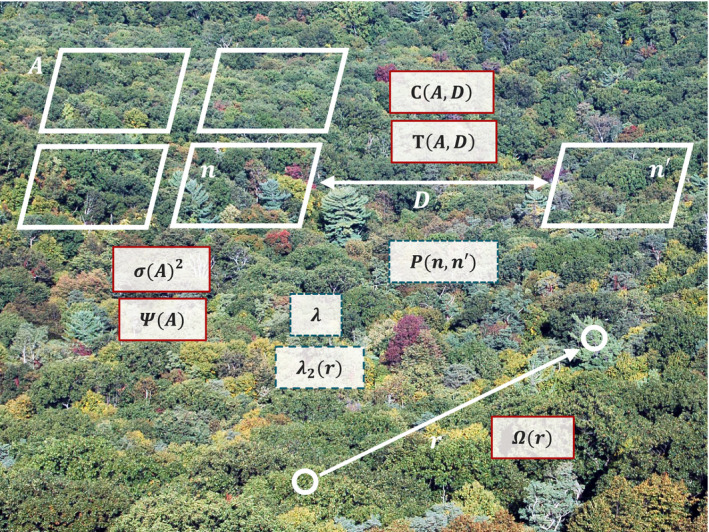
Graphical illustration of five focal spatial metrics, three intermediate statistics and supporting variables. The five focal spatial metrics are shown in solid red boxes near multiple plots of area A containing n individuals each (*σ*(*A*)^2^, Ψ(*A*)), pairs of plots of area A separated by distance D containing n and *n′* individuals respectively (C(A,D), T(A,D)), or pairs of locations separated by distance r that may each either contain an individual or not (Ω(r)). Three intermediate statistics used to calculate these metrics are indicated in dashed blue boxes: the first‐order intensity λ, the second‐order intensity function $\lambda_{2}\left(r\right)$ and the quadrat count distribution *P (n, n′)*. Image Credit: Shenandoah National Park, Virginia. Alexandra Fries, Integration and Application Network, University of Maryland Center for Environmental Science (ian.umces.edu/imagelibrary/)

The focus of this manuscript is on the relationship between five single‐species spatial metrics: the occupancy area curve, Taylor's Law, the neighborhood density function, the two‐plot variant of Taylor's Law and two‐plot turnover. The mathematical relationships between these five metrics are shown in summary form in Figure 1, with equations defining the exact relationships given below. The five spatial metrics, the intermediate statistics named above and other key variables are also illustrated graphically in Figure 2.

The five spatial metrics are linked in part by the three intermediate statistics described above (Figure 1). Beginning with the second‐order intensity function, the neighborhood density function or O‐ring, Ω(r), can be calculated as (Wiegand & Moloney, 2013)

(1) $$\Omega \left(r\right)=\frac{\lambda_{2}\left(r\right)}{\lambda }$$

Several other related spatial metrics, including the pair correlation function and Ripley's K, can also be easily calculated from the second order intensity (Wiegand & Moloney, 2004, 2013).

The second‐order intensity can then be linked to the quadrat count distribution as follows. First, the expected number of pairs of points in a plot, E[R], can be expanded to E[R] = E[*n*(*n*–1)] = E[*n* ^2^]–E[*n*]=σ^2^–*μ* + *μ* ^2^ where n is a random variable representing the number of points in a plot and μ and *σ^2^* are the mean and variance of the quadrat count distribution. Second, the quantity E[R] can also be calculated as a double integral of the second‐order intensity function over all possible locations of points in the plot (see Supporting Information 1). Following the notation of Diggle (2013), the relationship between the variance of the quadrat count distribution and the second‐order intensity is then

(2) $$\sigma {\left(A\right)}^{2}‐\mu (A)+\mu {\left(A\right)}^{2}=\int_{L}\int_{L}\lambda_{2}(r)dx_{1}dx_{2}$$

where L is the region covered by a plot of area A, $dx_{1}$ and $dx_{2}$ are two discs located at $x_{1}$ and $x_{2}$ within L, r is the distance between the two discs and the integrals are over all possible locations within L (Diggle, 2013). This equation can be used to calculate Taylor's Law, which relates *σ(A)^2^* to μ(A).

Extending the second‐order intensity to the case of two disjoint plots, a two‐plot variant of Taylor's Law can be defined as the covariance, C(A,D), in abundance between two plots as a function of both plot area A and the distance between the plots D. The relationship between the two‐plot Taylor's Law and the second‐order intensity can be derived using similar logic to the above as (Diggle, 2013)

(3) $$C(A,D)+\mu {\left(A\right)}^{2}=\int_{L}\int_{L^{\prime }}\lambda_{2}(r)dx_{2}dx_{2}$$

where L and *L′* are the regions covered by two plots, both of area A, and other definitions are as above. Here $x_{1}$ is constrained to lie in L and $x_{2}$ is constrained to lie in *L′*.

Turning finally to plot occupancy, the probability that a single plot of area A is occupied, Ψ(A), is given by

(4) Ψ(A)=1‐P(n=0|A)

where P(n=0|A) is the probability of a plot of area A containing zero individuals, which is given by the quadrat count distribution. The relationship between the probability of plot occupancy and area is known as the occupancy area curve (He & Condit, 2007).

Similar to the Sorensen index of community turnover or distance decay (Soininen et al., 2007), two‐plot turnover for a single species can be defined as the probability that two plots of area A separated by distance D are simultaneously occupied, divided by the probability of a single plot being occupied,

(5) $$T\left(A,D\right)=\frac{P(n>0,n^{\prime }>0|A,D)}{P(n>0|A)}=2+\frac{P(n=0,n^{\prime }=0|A,D)‐1}{\Psi \left(A\right)}$$

where *P(n = 0, n′ = 0|A,D)* is the joint probability that both plots are empty. This turnover metric can also be interpreted as the conditional probability that one plot is occupied, given that the other is occupied.

The first assumption required to use this framework, as described above, is that the point pattern defining individual locations is orderly, homogenous and isotropic. A second required assumption is a parametric form of the quadrat count distribution, which is needed to predict univariate and bivariate probabilities of occupancy. The framework as presented here requires a bivariate quadrat count distribution that can be parameterized with a vector of means and a covariance matrix, but which can otherwise take any parametric form. The univariate distribution for a single plot is then the marginal distribution of this bivariate distribution.

We follow previous investigators (Green & Plotkin, 2007; He & Legendre, 2002) in assuming a negative binomial distribution for the quadrat count distribution. The standard bivariate negative binomial distribution, however, does not allow for the specification of an arbitrary covariance independent of the mean and variance of the marginal distributions (Dunn, 1967; Johnson et al., 1997). As such, we construct an alternative bivariate negative binomial distribution as the sum of three independent underlying negative binomial distributions, which describe the sizes of a population of individuals occurring in the first plot independently of the second, a population of individuals occurring in the second plot independent of the first and a population of individuals with identical size occurring in both plots (Supporting Information 1).

In the results below, units are defined such that λ=1, or equivalently such that a plot of area 1 has an expected abundance of 1 individual, which ensures that μ(A)=A (see Supporting Information 1). As such, variation in plot area is equivalent to variation in expected abundance within a plot of fixed physical area.

### Theoretical examples

As described above, this unified framework can be used to derive any four of the five spatial metrics given knowledge of the fifth. We demonstrate this mathematical relationship with several concrete theoretical examples, in which the exact form of one metric is assumed and the forms of the remaining four metrics are derived.

We first consider the case where a species exhibits a power law form of Taylor's Law, which is well‐established both in space and time (Xiao et al., 2015) and takes the form

(6) $$\sigma {\left(A\right)}^{2}=a\mu {\left(A\right)}^{b}$$

where a is a constant often in the range of 0.1 to 100 and b is a constant commonly ranging from 1 to 2 (see Xiao et al., 2015 for examples and plots). We consider a one‐dimensional case, which might represent a length of a river or a time sequence, as well as a two dimensional case.

We next consider a community for which the second‐order intensity function takes the form of a Gaussian function, such that

(7) $$\lambda_{2}(r)=\alpha e^{‐\beta r^{2}}+1$$

A Gaussian second‐order intensity function is a particularly common assumption in point pattern analysis, as several Poisson cluster models (Wiegand & Moloney, 2013) have a second‐order intensity of this form (see below).

Finally, we calculate the shapes of all five spatial metrics in the case of complete spatial randomness (CSR). CSR can be understood as a limiting case of the examples above in which a=1 and b=1 or α=0.

Expressions for Taylor's Law, the neighborhood density function and the two‐plot Taylor's Law variant for four theoretical examples are shown in Table 1. These expressions can be combined with the general equations for the occupancy area curve (He & Gaston, 2003)

(8) $$\Psi \left(A\right)=1‐{\left(\frac{A}{\sigma {\left(A\right)}^{2}}\right)}^{\frac{A^{2}}{\sigma {\left(A\right)}^{2}‐A}}$$

and the equation for two‐plot turnover

(9) $$T\left(A,D\right)=2+\frac{1}{\Psi \left(A\right)}\left[{\left(\frac{A}{\sigma {\left(A\right)}^{2}}\right)}^{\frac{A^{2}(2\sigma {\left(A\right)}^{2}‐C(A,D))}{\sigma {\left(A\right)}^{2}(\sigma {\left(A\right)}^{2}‐A)}}‐1\right]$$

to calculate these remaining two metrics for each theoretical example.

**TABLE 1:** Equations for Taylor's Law, the neighborhood density function and two‐plot Taylor's Law variant for four theoretical examples

Assumed metric	*σ(A)^2^*	Ω(r)	C(A,D)
Power law Taylor's Law (one‐dimension)	$aA^{b}$	$\frac{1}{2}ab\left(b‐1\right)r^{b‐2}+1‐\delta \left(r\right)$	$\frac{1}{2}a\left[D^{b}‐2{\left(D+A\right)}^{b}+{\left(D+2A\right)}^{b}\right]$
Power law Taylor's Law	$aA^{b}$	$\approx {\mathrm{cr}}^{2(b‐2)}+1‐\frac{\delta (r)}{\pi r}$	$\approx cA\left(\frac{1}{\left(\gamma ‐1\right)\left(\gamma ‐2\right)}\left(D^{2‐\gamma }‐2{\left(D+\sqrt{A}\right)}^{2‐\gamma }+{\left(D+2\sqrt{A}\right)}^{2‐\gamma }\right)‐\right.\left.\frac{A}{12\left(\gamma +1\right)}\left(D^{‐\gamma }‐2{\left(D+\sqrt{A}\right)}^{‐\gamma }+{\left(D+2\sqrt{A}\right)}^{‐\gamma }\right)\right)$
Gaussian second‐order intensity	$\frac{\alpha }{\beta^{2}}[\sqrt{\pi \beta A}$ erf $\left(\sqrt{\beta A}\right)+e^{‐\beta A}{‐1]}^{2}+A$	$\alpha e^{‐\beta r^{2}}+1$	$\frac{\alpha \sqrt{\pi }}{2\beta^{3/2}}\left[\sqrt{\pi \beta A}\;\text{erf}\left(\sqrt{\beta A}\right)+e^{‐\beta A}‐1\right]\left[D\;\text{erf}\left(\sqrt{\beta }D\right)‐2\left(D+\sqrt{A}\right)\;\text{erf}\left(\sqrt{\beta }D+\sqrt{\beta A}\right)+\left(D+2\sqrt{A}\right)\;\text{erf}\left(\sqrt{\beta }D+2\sqrt{\beta A}\right)+\frac{1}{\sqrt{\pi \beta }}\left(e^{‐\beta D^{2}}‐2e^{‐\beta {\left(D+\sqrt{A}\right)}^{2}}+e^{‐\beta {\left(D+2\sqrt{A}\right)}^{2}}\right)\right]$
Complete spatial randomness	A	1	0

In each example, the form of one spatial metric is assumed and the remaining spatial metrics are derived (see Supporting Information 1). The remaining two metrics not shown in this table, the occupancy area curve and single‐species turnover, are calculated in the same manner for each example using the equations in the main text. All metrics apply to a landscape with units of length scaled so that λ=1. δ(r) is the Dirac delta function, which is equal to 1 when r=0 and zero elsewhere, and the constant $c=\frac{ab(b‐1)(2b‐1)}{\pi (2b‐1)‐4(b‐1)}$. The ≈ symbol indicates an approximation (see Supporting Information 2). Unless otherwise specified, all metrics are calculated for a two‐dimensional landscape.

**FIGURE 3:**
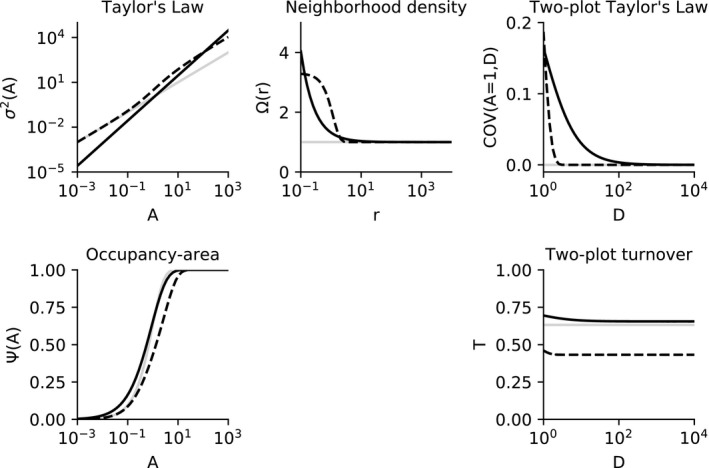
Example shapes of five spatial metrics calculated for three theoretical examples. In each example, the shape of one of the five metrics is assumed and the remaining four metrics are calculated using equations and parameters described in the text. Examples are Taylor's power law in two dimensions (black solid line), a Gaussian second‐order intensity function (dashed line) and complete spatial randomness (grey solid line). See Table 1 and main text for equations. Subplots showing each of the five metrics are located in the same relative positions as in Figure 1

Figure 3 illustrates the two‐dimensional examples in Table 1 graphically for the particular parameter values of a=0.88 and b=1.51 for the Taylor's power law examples, reflecting the median values of these parameters from a recent review (Xiao et al., 2015, Xiao Xiao *pers comm*) and α=2.30 and β=0.71 for the Gaussian second‐order intensity example, which are equal to the median values of these parameters across 171 species in the Barro Colorado Island dataset (see below). For the two‐plot Taylor's Law and turnover, the metrics are shown as a function of distance D for a fixed plot area A=1.

### Simulation study

We next test our mathematical framework by using a Thomas process model (Morlon et al., 2008; Wiegand & Moloney, 2013) to generate simulated species distributions (see Supporting Information 3 and 4). The Thomas process is a Poisson cluster model in which cluster centres are located by a Poisson process with intensity ρ, the number of individuals per cluster is drawn from a Poisson distribution with mean μ and the location of individuals within a cluster is defined by a bivariate normal distribution with variance *σ^2^* around a cluster center.

The pair correlation function of the Thomas process, which is equal to the second‐order intensity $\lambda_{2}\left(r\right)$ in the scaled units used in our analysis (see Results), is given by the Gaussian function (Wiegand & Moloney, 2013)

(10) $$\lambda_{2}\left(r\right)=\frac{\exp(‐r^{2}/4\sigma^{2})}{4\pi \rho \sigma^{2}}+1$$

In the notation above, $\alpha =1/4\pi \rho \sigma^{2}$ and $\beta =1/4\sigma^{2}$ for this particular point process model.

We simulate point patterns for each of three idealized ‘species’. We chose parameters for each idealized species based on empirical data from the Barro Colorado Island (BCI) tropical forest plot (see below). We began by dividing the species at BCI into three abundance tertiles. We fitted Thomas process models to each BCI species using the *thomas*.*pcf* function in the *spatstat* package in R (Baddeley et al., 2015; R Core Team, 2020), and then extracted the median values of landscape‐wide abundance n, α and β for low (n=108, α=4.5, β=3.8), medium (n=432, α=2.7, β=0.8) and high (n=1856, α=1.0, β=0.1) idealized species. These parameters were used to simulate 200 replicate point patterns for each idealized species in a 500 m by 1000 m landscape, matching the dimensions of the BCI plot.

For each simulation, we directly evaluate the shape of all five spatial metrics. An intermediate‐sized small plot of side length 25 m is used for calculations of two‐plot Taylor's Law and turnover, which are evaluated as functions of interplot distance D holding plot area constant. For each idealized species, we then calculate the median value of the metric at each A or D across all 200 simulations and use this median metric for comparison to theoretical predictions.

We generated theoretical predictions for each metric by using the equations derived for a species exhibiting a Gaussian second‐order intensity function, along with known values of α and β from the simulations. In reviewing the simulated data, however, we found that the median fitted α and β parameters or each idealized species deviated slightly from the target values for the low (α=5.1, β=4.8) and medium (α=2.9, β=0.9) species. We used these recovered parameter values to generate predictions for these idealized species.

Boswell and Patil (1970) note that under some conditions, the negative binomial is a good approximation for the quadrat count distribution in a Poisson cluster model with a Gaussian neighborhood density function. However, as this relationship is not exact, we expect greater deviation between simulations and predictions for the occupancy area curve and turnover metric, the two spatial metrics that rely on the assumption of a negative binomial quadrat count distribution.

**FIGURE 4:**
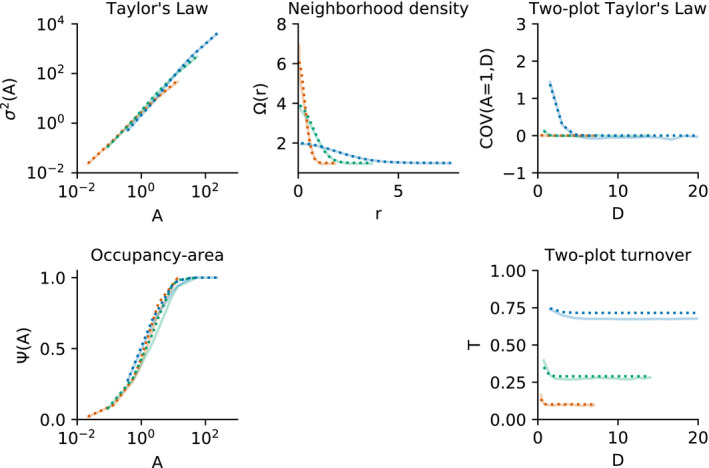
Results of simulation study. Each subplot plots one spatial metric for Thomas process simulations of low (orange), medium (green) and high (blue) idealized species. Light colored solid lines show median values of the metric for each species calculated directly from 200 replicate simulations, and dark dotted lines show the theoretical predictions for each metric based on known simulation parameters. Larger deviations between simulations and theoretical predictions for the occupancy area curve and turnover metrics reflect violations of framework assumptions for these metrics (see text)

Figure 4 shows the results of this simulation study, which demonstrate that the median values of Taylor's Law, the neighborhood density function and two‐plot Taylor's Law from the simulated data very closely match the associated theoretical predictions for all three idealized species. For the occupancy area curve and turnover, the theoretical predictions deviate more from the empirical results, as expected given that the negative binomial quadrat count distribution is only an approximation for this point process model. In particular, the theoretical predictions systematically overestimate the values of both metrics for all three idealized species, indicating that the simulated point patterns are more spatially aggregated than predicted by this theoretical framework. When considering variation across simulations within a species (Figure S1 in Supporting Information 1), the three idealized species have clearly distinguishable shapes of the neighborhood density function, two‐plot Taylor's Law and turnover metrics. Simulated Taylor's Law and occupancy area curves, in contrast, are largely overlapping across species, indicating that it would be difficult in practice to use the shape of these two metrics to infer the shape of the other three metrics for this particular point process model and parameter choices.

### Empirical study

Finally, we test the applicability of our framework to an empirical data set using data from the Barro Colorado Island tropical forest plot (Condit et al., 2019). Using the 2015 census, we consider 171 tree species with 70 or more living individuals (see Wiegand & Moloney, 2013). We directly measure the shapes of all five spatial metrics for each species, using a square plot with a 25 m side length for metrics involving both plot area and interplot distance. For each species and metric, we then fit the corresponding equation for that metric for a species with a Gaussian second‐order intensity, which gives one estimated value of α and β for each combination of species and metric (see Supporting Information 3 and 4).

A key result of the mathematical framework proposed here is that the five spatial metrics for any species are related in a predictable manner. To test this assertion, we calculate Spearman's rank correlation coefficients for the α and β parameters estimated from each possible pair of spatial metrics for all species in the community. This coefficient measures the strength of the linear relationship between the ranks of species’ parameter values within the community as estimated from two spatial metrics. Under the null hypothesis in which the shapes of two spatial metrics are independent of each other, this coefficient would be statistically insignificant and near zero. In contrast, a positive and statistically significant coefficient for any two spatial metrics indicates that there is a correlation between parameter values calculated using those two metrics, as would be expected if the mathematical framework is able to use the shape of one spatial metric to predict the shape of the other.

**FIGURE 5:**
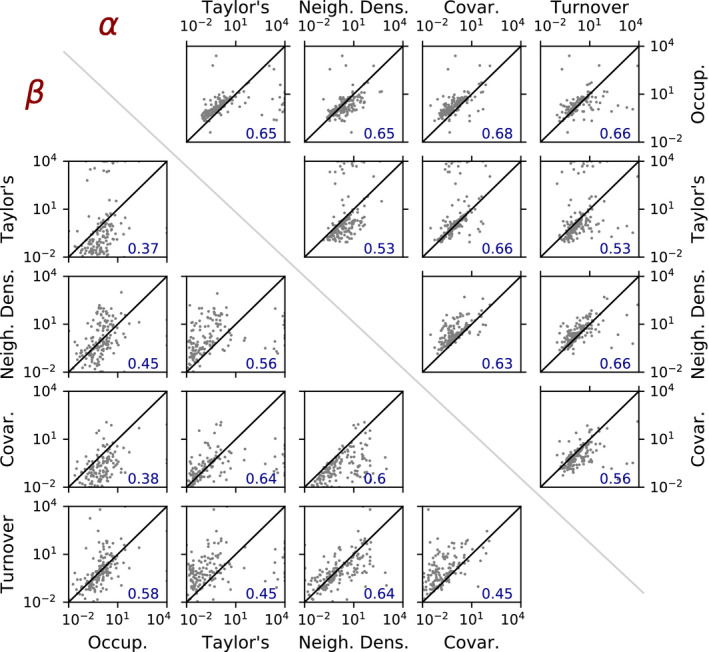
Results of empirical analysis of Barro Colorado Island data. Each subplot shows the correlation between the α (top right) or β (bottom left) parameters fitted from a pair of spatial metrics for 171 tree species at Barro Colorado Island. Blue text in each subplot gives the Spearman's rank correlation coefficient for each pair of metrics. *P*‐values for all Spearman's coefficients are <1e‐13 for α and <1e‐6 for β. The scatter in these pairwise correlation plots can be attributed in part to the observation that a Gaussian second‐order intensity is only an approximation for the spatial distributions of many of the BCI species (compare to variation observed in Morlon et al., 2008)

Figure 5 shows results from this empirical study using data from the Barro Colorado Island tropical forest plot. These results show that fitted values of α and β have relatively strong, positive and statistically significant correlations across all possible pairs of metrics, indicating that the equations predicted by this framework can be used to identify biologically and statistically significant relationships between the shapes of the spatial metrics (P‐values for all Spearman's ρ are <1e‐13 for α and <1e‐6 for β). As units for each species were scaled such that λ=1, these correlations do not reflect simply a variation in abundance between species. Overall, the correlations involving Taylor's Law and the two‐plot Taylor's Law are the weakest, suggesting that in practice, the shape of the remaining spatial metrics will not be as well‐predicted from knowledge of these particular metrics. Figures SI1.3–6 provide additional information on how variation in α and β parameters relate to the shapes of each of the five metrics.

### DISCUSSION

The framework above demonstrates the mathematical relationships between five important spatial metrics that are frequently considered separately. Expressing the quantitative relationships between these metrics requires two general assumptions. Deriving the relationship between Taylor's Law, the neighborhood density function and two‐plot Taylor's Law requires the assumption that the point pattern is orderly, homogeneous and isotropic. Relating these three metrics to single‐plot occupancy and two‐plot joint occupancy additionally requires the assumption of a parametric form for the bivariate quadrat count distribution, which we here assume to be negative binomial. As shown above, the ability to predict one metric from the known shape of another in practice will depend on several factors, including the extent to which a species’ point pattern meets these two assumptions, the sensitivity of the shapes of empirical metrics to changes in parameter values, which empirical metric is used to estimate parameters, and the ability to statistically recover accurate empirical parameter values from a point pattern.

The equivalence of area units and species density in the framework above highlights the additional observation that there is only a single, universal set of spatial metrics for any set of species that share one of the metrics. For example all species exhibiting a Taylor's power law with the same a and b parameters will exhibit identical parametric forms of all five spatial metrics, regardless of their density or abundance, with the exact numerical form of the metric scaled by a constant (see Supporting Information 1).

This mathematical relationship leads to at least four important implications for both basic and applied ecology. First, from the perspective of theory testing, this framework highlights that a theory that predicts one of the five metrics above also inherently makes predictions about the other four metrics. This provides an additional means of testing such theories with empirical data. As one concrete example, the Maximum Entropy Theory of Ecology predicts that the quadrat count distribution will take the form of a geometric distribution across spatial scales, a prediction that appears broadly consistent with empirical data (Harte, 2011; Harte & Newman, 2014; Harte et al., 2008). An application of the framework above, however, shows that this assumption leads to theoretically and empirically implausible forms of both the second‐order intensity function and the correlation in plot abundance with distance (Supporting Information 1).

Second, from the perspective of experimental design, this framework highlights that, at least in principle, data collected using one sampling scheme can be used to determine the shape of metrics that have traditionally been thought to require a different sampling approach. One useful translation would be to use nested measurements of plot abundance for a species, a type of data used to construct nested species–area relationships, to predict the second‐order intensity function or single‐species turnover, two metrics that otherwise require spatially explicit data to measure directly. The simulation study above suggests that this particular translation may be error‐prone given the overlap in Taylor's Law across parameter sets. In contrast, however, the empirical study suggests that among a large group of species, the shape of Taylor's Law does have significant predictive power in inferring the shape of other distance‐based spatial metrics.

Third, from the perspective of spatial ecology, the shape of the predicted single‐species turnover metrics highlights the potentially pervasive dominance of area over distance in determining spatial turnover. Although turnover is often analyzed in terms of the distance between plots, the equations above demonstrate that plot area may have a stronger influence on the value of thesemetrics than interplot distance. Figure SI1.2 shows that for the two‐dimensional Taylor's power law and Gaussian second‐order intensity examples, the turnover metric is more strongly influenced by plot area than by distance, particularly when the plot area is relatively large. Since the community‐level Sorensen index is the sum of the individual species turnover metrics, this result suggests that the shape of the species abundance distribution in a community may be the dominant influence on community turnover, rather than the distance between two plots. We note that this finding rests on the assumption that a species’ point pattern is homogenous, stationary and isotropic, an assumption that may break down at larger spatial scales where habitat heterogeneity and gradients become more prominent.

Finally, from the perspective of theory development, this framework lays the groundwork for future theories that can relate spatial metrics at the community level. For example, the single‐species framework presented here could be extended to the community level through the addition of a metacommunity species abundance distribution. Using the assumption of spatial independence between species that is common to many macroecological theories (see McGill, 2010), a species–area relationship can be constructed as the sum of occupancy area curves across species, and two‐plot community‐level turnover can be calculated as the sum of the single‐species turnover metric across species. This would allow for a new method for examining the general relationship between shapes of the species–area relationship and two‐plot turnover, a relationship that has been an important topic of research in spatial macroecology (Harte, 2011; Harte & Kinzig, 1997; Hui, 2009; McGlinn & Hurlbert, 2012; McGlinn et al., 2015; Tjørve & Tjørve, 2008). Similar community‐level metrics can be constructed in continuous space by summing second‐order intensities across species (Chave & Leigh, 2002).

Another community‐level expansion of this framework lies in the construction of a multi‐plot species–area relationship. To begin, note that the probability that a species is simultaneously absent from both of a pair of plots is part of the calculation of the two‐plot turnover metric above. This probability depends both on plot area and the distance between plots. As above, a metacommunity abundance distribution can be used with this probability to construct a species–area relationship for a pair of plots separated by a fixed distance as the area of either or both plots change. This logic can be further generalized to a landscape with more than two plots, where any given plot may change in area, where the network‐wide probability of presence is calculated using a vector of means and covariance matrix for the abundance of each species in the network. In this more general case of the species–area relationship, the change in species richness with the area will depend on the particular location within the network where the area is added or subtracted.

To support the expansion of this framework, several of the assumptions made above could be relaxed. The negative binomial quadrat count distribution could be replaced by other distributions that can be parameterized from plot mean, variance and covariance, such as the lognormal distribution (Limpert et al., 2001; Yue, 2002). The assumption of homogeneity could be relaxed by instead assuming that the observed point pattern is second‐order intensity‐reweighted stationary (Baddeley et al., 2000), a type of pattern that is created when an otherwise homogeneous pattern is thinned according to an intensity layer. Methods exist for recovering the underlying properties of, for example the second‐order intensity function from such a pattern given knowledge of the additional thinning layer (Baddeley et al., 2000; Wiegand & Moloney, 2013). The results from this second‐order intensity‐reweighted framework could be compared to those from the current framework in order to evaluate the importance of heterogeneity on the quantitative relationships between the five spatial metrics.

In summary, this work presents a mathematical framework demonstrating the relationships between five species‐level metrics of spatial pattern: the occupancy area curve, Taylor's Law, the neighborhood density function, a two‐plot variant of Taylor's Law and two‐plot turnover. This framework presents new avenues for theory testing, expands investigators’ options for spatial sampling and demonstrates the importance of plot area in driving patterns of turnover. The results also lay the groundwork for expanded, unified spatial theories that explicitly incorporate habitat heterogeneity and fragmentation at the community level.

## AUTHORSHIP

All authors designed and participated in developing the theoretical framework described here. J.K. and M.B. wrote the manuscript with contributions from K.W.

## Supporting information

Supplementary Material

Supplementary Material

Supplementary Material

Supplementary Material

## Data Availability

This manuscript presents largely theoretical research. Empirical data from Barro Colorado Island that are used for theory testing can be found on Dryad (https://doi.org/10.15146/5xcp‐0d46).
