# Supplementary material for: A unified framework for species spatial patterns: Linking the occupancy area curve, Taylor's Law, the neighborhood density function and two‐plot species turnover
Source: Ecol Lett. 2021 Aug 4;24(10):2043–53. doi: 10.1111/ele.13788 (PMC8518128; doi:10.1111/ele.13788)
Supplement: Supplementary file 1 — Supplementary Material [file ELE-24-2043-s003.pdf]

A unified framework for species spatial patterns: Linking the occupancy area curve, Taylor's Law, the neighborhood density function, and two-plot species turnover

Supporting Information 1

Justin Kitzes[1], Micah Brush[2], Kyle Walters[1]

1 - Department of Biological Sciences, University of Pittsburgh
2 - Department of Physics, University of California, Berkeley

1 Defining units of length and area

For notational convenience, and without loss of generality, we define units of length in our landscape such that the species of interest has a density of 1 individual per unit area. Under this definition, the first-order intensity $\lambda = 1$, and the expected number of individuals in a plot of area A is given by $\mu(A) = \lambda A = A$. Aside from notation, this approach also highlights that every species having a metric with the same parameters (for example, the a and b of Taylor's Law) is expected to show the same general form of the five spatial metrics, regardless of its abundance.

A point pattern with any original first-order intensity λ', as measured in a landscape in which length is measured in any original units, can be converted to a point pattern in which $\lambda = 1$ by multiplying all measures of length by the conversion factor $\sqrt{\lambda'}$. To verify this conversion, consider a square plot that contains an expected number of individuals $E[n]$. This expectation is invariant to the units in which lengths or distances are measured. In particular $E[n] = h'^2\lambda' = h^2\lambda$, where the superscript $'$ refers to side length and density measured in original units and the non-superscript terms refer to side length and density measured in scaled units. Since in scaled units $\lambda = 1$, it is clear that multiplying the original arbitrary length h' by $\sqrt{\lambda'}$ gives distances measured in scaled units h that ensure that $\lambda = 1$.

For a concrete example, consider an original landscape in which $\lambda' = 4$. A square plot of side length $h' = 2$ has $E[n] = h'^2\lambda' = 16$. Multiplying all distances in the landscape by the factor $\sqrt{\lambda'} = 2$ gives a square plot of side length $h = 4$ in this scaled landscape. In this same plot with distances measured in scaled units, $E[n] = 16 = h^2\lambda = 16\lambda$ and thus $\lambda = 1$, as per the goals of the unit conversion.

Although this conversion is convenient for notational purposes, the validity of the framework described below does not depend on it. To construct or apply the equations below in original, unscaled units, simply replace all occurrences of the variable A with $\lambda' A'$ and all occurrences of the variable h with $h'\sqrt{\lambda'}$, where the superscript $'$ refers to areas and lengths measured in original units.

An additional notational convenience of this unit conversion is that for a landscape where $\lambda = 1$, the neighborhood density function $\Omega(r)$ is identical to the second-order intensity function, $\lambda_2(r)$. Below, we derive results for the second-order intensity function and report these as the results for both $\Omega(r)$ and $\lambda_2(r)$ in the main manuscript.

2 Relationship between plot mean and variance and second-order intensity function

One of the key expressions in this manuscript is the relationship between plot variance and the integral of the second-order intensity function (Illian *et al.* 2008; Diggle 2013). This relationship can be understood intuitively by considering the expected number of pairs of points found in a plot.

Consider a single plot of any area and geometry containing some number of individuals of a species, with the number of individuals represented by the random variable n. Define the expected number of unordered pairs of points in this plot as $E[R]$. From basic rules of expectations, we have

$$E[R] = E[n(n-1)] = E[n^2] - E[n] = \sigma^2 - \mu + \mu^2 \tag{1}$$

where μ and σ^2 are the mean and variance, respectively, of n.

Next, within this same plot, consider two infinitesimally small discs of area dx_1 and dx_2, which are small enough that

they can only contain zero or one individual. Imagine moving each of these two discs to every possible location within the plot. For each possible pair of locations for the two discs, the discs are separated by a distance r. The probability of encountering an individual in both discs simultaneously, which is equivalent to the expected number of pairs of individuals in the pair of discs, is then equal by definition to $\lambda_2(r)$. By integrating over all possible locations for each disc, we can thus also calculate the expected number of unordered pairs of individuals in the plot as

$$E[R] = \int_L \int_L \lambda_2(r)\, dx_1\, dx_2 \tag{2}$$

where L refers to the region of the plot. In a two-dimensional case, for example, the integral above can be written as a quadruple integral over the x and y coordinates of the two discs.

Equating the two equations above leads to the relationship between the mean and variance of the quadrat count distribution and the integral over the second-order intensity function that is described in the main manuscript. Similar logic leads to the expression for the covariance in abundance across two plots as an integral of the second-order intensity function, with the only difference being that the discs dx_1 and dx_2 are constrained to be located in two different plots.

In practice, the most substantial challenge in applying this framework is evaluating the integrals, or in some cases solving the integral equations, resulting from the above. One approach is direct integration, in which the integral is taken over the coordinate locations of the discs dx_2 and dx_2. As this leads to a double integral for one-dimensional landscapes or a quadruple integral for two-dimensional landscapes, analytic solutions do not appear to be available for many cases. A second approach is based on the line-line picking distribution, which gives the probability that a randomly selected pair of locations (not individuals or points of a point pattern) within a geometrical shape will be separated by a distance r (Mathai 1999). By multiplying these probabilities by the total number of possible locations for the two discs, the number of pairs of locations at each distance r can then be calculated. The multiple integrals resulting from the above can then be expressed as a single integral over the set of allowable distances r between locations at which a point might appear. Both approaches are used in the derivations below.

3 The univariate negative binomial distribution and the occupancy area curve

For a single plot of area A, we assume that the quadrat count distribution follows a negative binomial distribution. The negative binomial distribution is given by

$$P(n) = \binom{n+k-1}{n} p^n (1-p)^k \tag{3}$$

where n is the abundance of a plot and k (often written as r in a non-ecological context) is interpretable as an aggregation parameter.

For describing occupancy, we are specifically interested in the case of $n = 0$, which occurs with the probability

$$P(n = 0) = (1-p)^k \tag{4}$$

The mean and variance of the negative binomial distribution are given by $\mu = pk/(1-p)$ and variance $\sigma^2 = pk/(1-p)^2$, leading to the alternative parameterization

$$P(n = 0) = \left(\frac{\mu}{\sigma^2}\right)^{\frac{\mu^2}{\sigma^2-\mu}} \tag{5}$$

The occupancy area curve, $\Psi(A)$, is then defined as (He & Gaston 2003)

$$\Psi(A) = 1 - P(n = 0|A) = 1 - \left(\frac{\mu(A)}{\sigma(A)^2}\right)^{\frac{\mu(A)^2}{\sigma(A)^2-\mu(A)}} \tag{6}$$

Since $\mu(A) = A$ when $\lambda = 1$, as described above, this simplifies to

$$\Psi(A) = 1 - \left(\frac{A}{\sigma(A)^2}\right)^{\frac{A^2}{\sigma(A)^2 - A}} \tag{7}$$

This general equation can be used to calculate the occupancy area curve for all theoretical examples in the main text, given knowledge of Taylor's Law for that particular example.

4 The bivariate negative binomial distribution

As described in the main text, the traditional form of the bivariate negative binomial distribution (Johnson *et al.* 1997) does not allow for arbitrary specification of positive correlation. We thus define an alternative bivariate negative binomial distribution based on an additive combination of three independent underlying negative binomial distributions, as described below.

Presume that we have known values for the mean μ, variance σ^2, and covariance COV in abundance for a pair of plots of area A and distance between plots D. Based on these known values, our specific goal is to determine $P(n > 0, n' > 0)$, the joint probability that both plots in a pair are occupied. This expression, along with a known expression for $\Psi(A)$, determines two-plot turnover.

Define the random variable n as the abundances of a species in the first of a pair of plots (all results below apply symmetrically to the second plot in the pair, with abundance n'). When considering only this single plot in isolation, n represents a draw from a negative binomial quadrat count distribution with mean μ and variance σ^2. We consider the random variable n to represent a sum of two underlying random variables, n_S and n_L. Both n_S and n_L are negative binomial distributed and are independent of each other. We then have

$$\begin{aligned} n = n_S + n_L &\sim \text{NB}(k = k_S + k_L, p) \\ n_S &\sim \text{NB}(k_S, p) \\ n_L &\sim \text{NB}(k_L, p) \end{aligned} \tag{8}$$

We interpret the random variable n_S as a set of individuals in a plot that result from a "small scale" population process (birth, death, and dispersal) that applies only to that plot, independent of the second plot of the pair. This includes, for example, population dynamics internal to the plot as well as short-distance dispersal. The random variable n_L, in contrast, represents "large scale" population processes that result in an identical number of individuals being placed in each plot in the pair. The total abundance of a plot represents the contributions of these small-scale and large-scale processes, which operate independently of each other. Note that aside from this mechanistic basis for Eq. 8, this equation can also be interpreted purely phenomenologically as a means of specifying varying degrees of correlation in abundance between two plots.

Equation 8 provides a simple and convenient means of specifying arbitrary correlations between the abundances in two plots based on the relative contributions of n_S and n_L to n. In the limit $E[n_S] \to E[n]$ and $E[n_L] \to 0$, the abundance of the two plots n and n' are independent of each other, as the abundance of each plot reflects independent draws of n_S. In the limit $E[n_L] \to E[n]$ and $E[n_S] \to 0$, both plots will have an identical abundance and hence a correlation of 1.

The probability that one plot is unoccupied is once again given by

$$P(n = 0) = (1 - p)^k \tag{9}$$

The probability that both plots are jointly unoccupied, $P(n = 0, n' = 0)$, is given by the product of the probabilities that $n_S = 0$ in the first plot, $n'_S = 0$ in the second plot, and $n_L = 0$ (since these three random variables are all independent of each other). This leads to the expression

$$P(n = 0, n' = 0) = (1 - p)^{k_S}\,(1 - p)^{k_S}\,(1 - p)^{k_L} = (1 - p)^{2k_S + k_L} \tag{10}$$

The next step is to determine the values of the three unknown parameters k_S, k_L and p in terms of the mean μ, variance σ^2, and covariance COV in plot abundance. Considering first the single plot abundance n, the parameters p and k are given by

$$p = \frac{\sigma^2 - \mu}{\sigma^2} \tag{11}$$

$$k = \frac{\mu^2}{\sigma^2 - \mu} \tag{12}$$

The next task is to solve for k_S and k_L. The covariance between the abundance in the first plot, $n = n_S + n_L$, and the abundance in the second plot, $n' = n'_S + n_L$ (note that the same variable n_L appears in both expressions), can be expressed as

$$\mathrm{COV}(n = n_S + n_L, n' = n'_S + n_L) = \mathrm{COV}(n_S, n'_S) + \mathrm{COV}(n_S, n_L) + \mathrm{COV}(n_L, n'_S) + \mathrm{COV}(n_L, n_L) \tag{13}$$

Because n_S, n'_S and n_L are independent random variables, Eq. 13 reduces to

$$\mathrm{COV}(n, n') = \mathrm{COV}(n_L, n_L) = \sigma_L^2 \tag{14}$$

As the variance of a negative binomial distribution is $\sigma_L^2 = pk_L/(1-p)^2$, we can use the relationship $\mathrm{COV} = \sigma_L^2$ to solve for k_L as

$$k_L = \frac{\mathrm{COV}(n, n')(1-p)^2}{p} = \frac{\mu^2\,\mathrm{COV}(n, n')}{\sigma^2(\sigma^2 - \mu)} \tag{15}$$

Since $k = k_S + k_L$, we then have

$$k_S = k - k_L = \frac{\mu^2}{\sigma^2 - \mu} - \frac{\mathrm{COV}(n, n')\,\mu^2}{\sigma^2(\sigma^2 - \mu)} = \frac{\mu^2(\sigma^2 - \mathrm{COV}(n, n'))}{\sigma^2(\sigma^2 - \mu)} \tag{16}$$

The probability that both plots are jointly unoccupied can then be expressed as

$$P(n = 0, n' = 0) = \left(\frac{\mu}{\sigma^2}\right)^{\frac{\mu^2(2\sigma^2 - \mathrm{COV}(n,n'))}{\sigma^2(\sigma^2 - \mu)}} \tag{17}$$

Finally, considering the equation above across multiple plot scales, we have $\mu = A$ under the unit definition above and replace $\mathrm{COV}(n, n')$ with the function notation $\mathrm{C}(A, D)$, which expresses that the covariance between n and n' will depend on the values of A and D for the pair of plots. This gives

$$P(n = 0, n' = 0 | A, D) = \left(\frac{A}{\sigma(A)^2}\right)^{\frac{A^2(2\sigma(A)^2 - \mathrm{C}(A,D))}{\sigma(A)^2(\sigma(A)^2 - A)}} \tag{18}$$

This general equation can be used to calculate two-plot turnover for all examples in the main text, given knowledge of Taylor's Law and the two-plot variant of Taylor's Law for that particular example.

5 The two-plot turnover metric

The Sorensen index of community turnover or distance decay is a widely-used metric for describing two-plot turnover in communities. This index is calculated as twice the number of species shared in common between two plots divided by the sum of the number of species in each plot. When predicted using theory, the index can be defined in terms of expectations as

$$S = \frac{2E[U]}{E[X] + E[Y]} \tag{19}$$

where $E[U]$ is the expected number of species shared between the two plots and $E[X]$ and $E[Y]$ are the expected number of species found in each of the two plots. When the two plots are of the same area and $E[X] = E[Y]$, this equation reduces to $S = E[U]/E[X]$, which is simply the expected number of species shared between the two plots divided by the expected number of species in a single plot. Note that S will likely be dependent both on the area of the two plots and the distance between them.

Analogously for a single species, we can define a turnover metric T as the joint probability of the species occurring in both plots divided by the probability of the species occurring in a single plot.

$$\mathrm{T}(A, D) = \frac{P(n > 0, n' > 0|A, D)}{P(n > 0|A)} \tag{20}$$

For the four possible combinations of species occupancy across the two plots, we have

$$P(n > 0, n' > 0) + P(n > 0, n' = 0) + P(n = 0, n' > 0) + P(n = 0, n' = 0) = 1 \tag{21}$$

Because of the symmetry between n and n' when both plots are the same area, this simplifies to

$$P(n > 0, n' > 0) = 1 - 2P(n > 0, n' = 0) - P(n = 0, n' = 0) \tag{22}$$

From the marginal probability for a single plot, we also have

$$P(n = 0) = P(n = 0, n' > 0) + P(n = 0, n' = 0) \tag{23}$$

Substituting Eq. 22 and Eq. 23 into Eq. 20 gives

$$\mathrm{T}(A, D) = \frac{1 - 2P(n = 0|A) + P(n = 0, n' = 0|A, D)}{P(n > 0|A)} \tag{24}$$

Recognizing that $\Psi(A) = P(n > 0|A) = 1 - P(n = 0|A)$, the above can be rearranged and simplified to

$$\begin{aligned} \mathrm{T}(A, D) &= \frac{2\Psi(A) + P(n = 0, n' = 0|A, D) - 1}{\Psi(A)} \\ &= 2 + \frac{P(n = 0, n' = 0|A, D) - 1}{\Psi(A)} \end{aligned} \tag{25}$$

6 Example: Power law Taylor's Law in one dimension

6.1 Taylor's Law

In this section, we consider the common power law form of Taylor's Law given by

$$\sigma(A)^2 = a\mu(A)^b \tag{26}$$

applied in a one-dimensional landscape. Such a one-dimensional landscape can describe either a spatial pattern, such as a species population arranged along a river, or a temporal pattern, in which case the "landscape" describes the appearence of individuals in a sample over time. This case provides a uniquely analytically tractable example, with solutions that can be compared to the approximate solutions for the two-dimensional case below. Note that in this one-dimensional case, A is defined as the length of a one-dimensional "plot," which is a line segment.

6.2 Neighborhood density function

Using the line-line picking approach (Mathai 1999), we have the general equation

$$\sigma^2(A) - A + A^2 = \int_0^A N(r)\lambda_2(r)dr \tag{27}$$

where $N(r)$ is the number of pairs of locations separated by distance r in the plot. Using Taylor's Law from Eq. 26 gives

$$aA^b - A + A^2 = \int_0^A N(r)\lambda_2(r)dr \tag{28}$$

The probability distribution describing the distance r between two randomly chosen locations (distinct from the distribution of distances between known individuals or points in a point pattern) on a line segment is known as the line-line picking distribution (Mathai 1999) and is given by

$$P(r) = \frac{2}{A} - \frac{2r}{A^2} \tag{29}$$

The total number of ordered pairs of locations in a line segment of length A is A^2, and thus the number of pairs of locations at each distance r is given by

$$N(r) = 2(A - r) \tag{30}$$

Substituting $N(r)$ into Eq. 28 gives the expression

$$\frac{1}{2}\left(aA^b - A + A^2\right) = \int_0^A (A - r)\lambda_2(r)dr \tag{31}$$

This is a Volterra integral equation of the first kind with a difference kernel, which can be solved for $\lambda_2(r)$. The solution is well-known to be the second derivative, with respect to A in this example, of the left hand side. The second order intensity is thus

$$\lambda_2(r) = \frac{1}{2}ab(b-1)r^{b-2} + 1 - \delta(r) \tag{32}$$

where $\delta(r)$ is the Dirac delta function, which is equal to 1 when $r = 0$ and equal to zero elsewhere. We assume the convention in which the Heaviside function $H(\theta)$, which is the integral of the delta function, has the value $H(0) = 1/2$.

6.3 Two-plot Taylor's Law

As described in the main text, the second-order intensity function is also related to the covariance in abundance between two non-overlapping plots, which we define as the two-plot variant of Taylor's Law. Define D as the distance between the closest edges of two square plots, where $D > 0$ to avoid plot overlap. In one dimension, the minimum possible distance between a point occurring in the first plot and a point occurring in the second plot is thus D, and the maximum possible distance is $D + 2A$. We can then write

$$\mathrm{C}(A, D) = \int_{D}^{D+2A} N(r)\lambda_2(r)dr - A^2 \tag{33}$$

The number of pairs of points located a distance of D or $D + 2A$ apart approaches zero as r approaches these limits. At $r = D + A$, there are A such pairs of points. This leads to a two-part expression for N(r),

$$N(R) = \begin{cases} r - D & \text{if } D \leq r \leq D + A \\ D + 2A - r & \text{if } D + A < r \leq D + 2A \end{cases} \tag{34}$$

which leads to the equation for $\mathrm{C}(A, D)$

$$\mathrm{C}(A, D) = \int_{D}^{D+A} (r - D)\left(\frac{1}{2}ab(b-1)r^{b-2} + 1\right)dr + \int_{D+A}^{D+2A} (D + 2A - r)\left(\frac{1}{2}ab(b-1)r^{b-2} + 1\right)dr - A^2 \tag{35}$$

Note that we have dropped the $\delta(r)$ term from Eq. 32 in the above, as the limits of integration above do not include $r = 0$ so long as $D > 0$. This integrates to

$$\mathrm{C}(A, D) = \frac{1}{2}a\left[D^b - 2(D + A)^b + (D + 2A)^b\right] \tag{36}$$

7 Example: Power law Taylor's Law in two-dimensions

7.1 Taylor's Law

In this section, we begin with the common power law form of Taylor's Law, once again given by

$$\sigma(A)^2 = a\mu(A)^b \tag{37}$$

Note that in this example, A represents a two-dimensional area. All plots are assumed to be square, with side length $h = \sqrt{A}$.

7.2 Second-order intensity function

Using the same line-line picking approach as in Example 1, adjusted for a square plot, we begin with the expression

$$\sigma(A)^2 - h^2 + h^4 = \int_0^{h\sqrt{2}} N(r)\lambda_2(r)dr \tag{38}$$

where $h = \sqrt{A}$ is the length of a side of a square plot of area A and $N(r)$ is the number of pairs of locations at distance r in the plot. The distribution $P(r)$ for a square is known (Mathai *et al.* 1999; Weisstein 2020), and multiplying this by $h^2h^2 = h^4$ possible pairs of locations gives

$$N(r) = \begin{cases} 2r\left(r^2 - 4rh + \pi h^2\right) & \text{if } 0 \leq r < h \\ 2r\left(4h\sqrt{r^2 - h^2} - r^2 + (\pi - 2)\,h^2 - 4h^2 \arctan\left(\frac{\sqrt{r^2-h^2}}{h}\right)\right) & \text{if } h \leq r \leq \sqrt{2}h \end{cases} \tag{39}$$

There does not appear to be a simple, exact solution for $\lambda_2(r)$ in the integral equation above. In the Supporting Information 2, we show that a very good approximation for $\lambda_2(r)$ in this two-dimensional case is

$$\lambda_2(r) \approx c r^{2(b-2)} + 1 - \frac{\delta(r)}{\pi r} \tag{40}$$

where the constant c is given by

$$c = \frac{ab(b-1)(2b-1)}{\pi(2b-1) - 4(b-1)} \tag{41}$$

As in the previous one-dimensional example, $\delta(r)$ is the Dirac delta function and we assume the Heaviside function has the value $H(0) = 1/2$.

7.3 Two-plot Taylor's Law

As it is difficult to directly generalize the line picking distribution given above for a single square to the case of two squares (Chu 2006), we take a two-part line picking approach to deriving the two-plot Taylor's Law.

Consider first two vertical line segments, each of height h and separated by distance d, standing with their bottom ends on the x-axis. The distribution of distances $P(r)$ between two randomly chosen points, one on each line segment, is given by (Mathai *et al.* 1999). The total number of pairs of points located on these line segments is

$$\int_d^{\sqrt{h^2+d^2}} 2r \left(\frac{h}{\sqrt{h^2 - d^2}} - 1 \right) \lambda_2(r) dr \tag{42}$$

A square plot can then be defined by "sweeping" a vertical line segment of height h across a segment of the x-axis of length h. The number of points shared between two square plots can thus be determined by integrating the number of points shared in the two vertical line segments across allowable locations on the x-axis of the two vertical segments, defined such that the left-most line segment must fall within the region of the left-most plot and the right-most line segment must fall within the region of the right-most plot. The integral over the allowable distances d between the vertical line segments is identical to the integral over pairs of locations in two line segments that is used to calculate the two-plot Taylor's Law in Example 1. By similar logic, we thus have the equation

$$\begin{aligned} \mathrm{C}(A, D) = & \int_D^{D+h} (d - D) \int_d^{\sqrt{h^2+d^2}} 2r \left(\frac{h}{\sqrt{r^2 - d^2}} - 1 \right) \lambda_2(r)\, dr\, dd + \\ & \int_{D+h}^{D+2h} (D + 2h - d) \int_d^{\sqrt{h^2+d^2}} 2r \left(\frac{h}{\sqrt{r^2 - d^2}} - 1 \right) \lambda_2(r)\, dr\, dd - A^2 \end{aligned} \tag{43}$$

Using the approximation for $\lambda_2(r)$ in Eq. 40, the value of $\mathrm{C}(A, D)$ can be calculated for any given plot area A and distance between plots D by numerical integration.

In Supporting Information 2, we derive an approximation for the above integral for cases in which $D > h$, such that plots are separated by a gap equal to at least the length of the side of a plot. This approximation is given by

$$\begin{aligned} \mathrm{C(A, D)} \approx cA \Bigg(& \frac{1}{(\gamma-1)(\gamma-2)} \left(D^{2-\gamma} - 2(D + \sqrt{A})^{2-\gamma} + (D + 2\sqrt{A})^{2-\gamma} \right) - \\ & \frac{A}{12(\gamma+1)} \left(D^{-\gamma} - 2(D + \sqrt{A})^{-\gamma} + (D + 2\sqrt{A})^{-\gamma} \right) \Bigg) \end{aligned} \tag{44}$$

8 Example: Gaussian second-order intensity

8.1 Second-order intensity function

In this section, we begin with the assumption of a Gaussian form of the second order intensity function, $\lambda_2(r)$, which is given by

$$\lambda_2(r) = \alpha e^{-\beta r^2} + 1 \tag{45}$$

8.2 Taylor's Law

Unlike the two examples above, we here use a direct integration approach to derive the remaining spatial metrics from the neighborhood density function. We begin by considering a single square plot of area A, with sides of length h, placed with its bottom left corner at the origin. The coordinates (x_1, y_1) and (x_2, y_2) represent the location of two small discs within the square. Defining the distance between the two discs as $r = \sqrt{(x_2 - x_1)^2 + (y_2 - y_1)^2}$ leads to the expression

$$\sigma^2(A) - A + A^2 = \int_0^h \int_0^h \int_0^h \int_0^h \lambda_2\left(\sqrt{(x_2 - x_1)^2 + (y_2 - y_1)^2}\right) dx_1 dx_2 dy_1 dy_2 \tag{46}$$

Substituting the Gaussian second order intensity function leads to

$$\sigma^2(A) - A + A^2 = \int_0^h \int_0^h \int_0^h \int_0^h \left(\alpha e^{-\beta[(x_2 - x_1)^2 + (y_2 - y_1)^2]} + 1\right) dx_1 dx_2 dy_1 dy_2 \tag{47}$$

Rearranging leads to

$$\sigma^2(A) - A + A^2 = \alpha \left[\int_0^h \int_0^h e^{-\beta(x_2 - x_1)^2} dx_1 dx_2\right]\left[\int_0^h \int_0^h e^{-\beta(y_2 - y_1)^2}\, dy_1 dy_2\right] + \int_0^h \int_0^h \int_0^h \int_0^h dx_1 dx_2 dy_1 dy_2 \tag{48}$$

which integrates to

$$\sigma^2(A) = \frac{\alpha}{\beta^2}\left[\sqrt{\pi\beta A}\,\mathrm{erf}(\sqrt{\beta A}) + e^{-\beta A} - 1\right]^2 + A \tag{49}$$

where $\mathrm{erf}(x)$ is the error function.

8.3 Two-plot Taylor's Law

The covariance in abundance between two plots can be evaluated in a similar fashion to the variance in a single plot. We have the general expression

$$\mathrm{C}(A, D) + A^2 = \int_0^h \int_{D+h}^{D+2h} \int_0^h \int_0^h \lambda_2\left(\sqrt{(x_2 - x_1)^2 + (y_2 - y_1)^2}\right) dx_1 dx_2 dy_1 dy_2 \tag{50}$$

where the right hand side differs from the previous calculation of single-plot variance only in the limits of integration for x_2. The variable D here represents the distance along the x-axis between the square in which the first disc and second disc are located. Using a Gaussian intensity function and following the same procedure as for single-plot variance leads to

$$\begin{aligned}
\mathrm{C}(A,D) =& \frac{\alpha\sqrt{\pi}}{2\beta^{3/2}}\left[\sqrt{\pi\beta A}\,\mathrm{erf}\left(\sqrt{\beta A}\right) + e^{-\beta A} - 1\right] \\
&\left[D\,\mathrm{erf}\left(\sqrt{\beta}D\right) - 2\left(D+\sqrt{A}\right)\,\mathrm{erf}\left(\sqrt{\beta}D+\sqrt{\beta A}\right) + \left(D+2\sqrt{A}\right)\,\mathrm{erf}\left(\sqrt{\beta}D+2\sqrt{\beta A}\right)\right. \\
&\left. + \frac{1}{\sqrt{\pi\beta}}\left(e^{-\beta D^2} - 2e^{-\beta(D+\sqrt{A})^2} + e^{-\beta(D+2\sqrt{A})^2}\right)\right]
\end{aligned} \tag{51}$$

9 Example: Complete spatial randomness

For comparison to the relationships above, we consider the form of the five spatial metrics in the case of complete spatial randomness (CSR), which is defined by the two equivalent statements that (a) the number of points in a plot of area A follows a Poisson distribution with mean λA, and (b) each point is randomly and independently located within the larger landscape (Diggle 2013). To demonstrate the quantitative relationship between the five spatial metrics for the case of CSR, we use definition (a) above, which defines both the occupancy area curve and Taylor's Law, and use this to derive the other three spatial metrics.

The quadrat count distribution under CSR takes the form of a Poisson distribution, which is the limiting form of the negative binomial distribution as $k \to \infty$ for a constant mean. We thus use equations for the Poisson distribution in this example where needed, rather than those for the negative binomial distribution given above.

9.1 Occupancy area curve

Under CSR, the quadrat count distribution is a Poisson distribution. In a landscape with scaled units such that $\lambda = 1$ and $\mu(A) = A$, we thus have

$$\Psi(A) = 1 - P(n=0|A) = 1 - e^{-\mu(A)} = 1 - e^{-A} \tag{52}$$

9.2 Taylor's Law

Since the variance and mean of the Poisson distribution are equal, Taylor's Law is given by

$$\sigma(A)^2 = \mu(A) = A \tag{53}$$

9.3 Second-order intensity function

Using Eq. 46 with $\sigma^2(A) = A$ and $A^2 = h^4$, we have

$$h^4 = \int_0^h \int_0^h \int_0^h \int_0^h \lambda_2\left(\sqrt{(x_2-x_1)^2+(y_2-y_1)^2}\right) dx_1 dx_2 dy_1 dy_2 \tag{54}$$

We can see that the solution to this equation is

$$\lambda_2(r) = 1 \tag{55}$$

which is consistent with the observation that points are randomly and independently distributed. In the more general case in which $\lambda \neq 1$, $\lambda_2(r) = \lambda^2$ under CSR.

9.4 Two-plot Taylor's Law

Using Eq. 50 and Eq. 55, we have

$$\mathrm{C}(A,D) + h^4 = \int_0^h \int_{D+h}^{D+2h} \int_0^h \int_0^h dx_1 dx_2 dy_1 dy_2 = h^4 \tag{56}$$

and thus

$$\mathrm{C}(A, D) = 0 \tag{57}$$

9.5 Two-plot turnover

Under random placement, the univariate quadrat count distribution is Poisson and the bivariate quadrat count distribution is the product of two independent Poisson random variables. As a result, $P(n = 0, n' = 0|A, D) = P(n = 0|A, D)P(n = 0|A, D)$, with $P(n = 0|A, D) = e^{-A}$ as noted above. The two-plot turnover metric from Eq. 24 thus becomes

$$\mathrm{T}(A, D) = \frac{1 - 2e^{-A} + e^{-2A}}{1 - e^{-A}} = \frac{(1 - e^{-A})^2}{1 - e^{-A}} = 1 - e^{-A} = \Psi(A) \tag{58}$$

This is once again consistent with the principle of CSR, as the conditional occupancy of a second plot given the occupancy of a first plot is equal to the unconditional occupancy of the second plot.

10 Application to Maximum Entropy Theory

The Maximum Entropy Theory of Ecology (METE) (Harte *et al.* 2008; Harte 2011; Harte & Newman 2014) is an ecological theory that predicts many well-known ecological patterns based on knowledge of a small number of state variables and the principle of maximizing information entropy (MaxEnt) (Jaynes 1982). Details of the theory can be found in the references above.

Here we focus on one specific prediction of the theory, which is that the quadrat count distribution, denoted $\Pi(n)$ in the theory, will take the form of a geometric distribution across all spatial scales. This result arises from a relatively straightforward application of MaxEnt to the problem of determining the probability that an urn will contain a given number of balls when only the mean number of balls per urn is known. The MaxEnt prediction for this problem, which is analogous to the quadrat count distribution in the spatial framework derived here, is the geometric distribution (Jaynes 1982; Harte *et al.* 2008). When applied in a spatial context in METE, this distribution is not scale dependent and is thus predicted to apply at all spatial scales, for all possible mean numbers of individuals per plot.

The relationship between the variance and mean of a univariate negative binomial quadrat count distribution is $\sigma^2 = \mu + \mu^2/k$. Using $k = 1$ to retrieve a geometric distribution and scaling units such that $\lambda = 1$ and hence $\mu = A$, the METE prediction for Taylor's Law is thus $\sigma(A)^2 = A + A^2$.

Using the approach of direct integration, the relationship between Taylor's Law and the second-order intensity function is given by Eq. 46. Substituting Taylor's Law under METE into the left hand side and recognizing that $A = h^2$ for a square plot gives

$$2h^4 = \int_0^h \int_0^h \int_0^h \int_0^h \lambda_2 \left(\sqrt{(x_2 - x_1)^2 + (y_2 - y_1)^2} \right) dx_1 dx_2 dy_1 dy_2 \tag{59}$$

This equation takes the same form as that for the case of complete spatial randomness (Eq. 54). The solution to this integral equation is

$$\lambda_2(r) = 2 \tag{60}$$

We then have the equation for covariance or two-plot Taylor's Law, which is

$$\mathrm{C}(A, D) + h^4 = \int_0^h \int_{D+h}^{D+2h} \int_0^h \int_0^h 2 dx_1 dx_2 dy_1 dy_2 \tag{61}$$

leading to

$$\mathrm{C}(A, D) = (2-1)\, h^4 = A^2 \tag{62}$$

METE thus predicts that the covariance in plot abundance is a positive value that depends on plot area but is constant with distance. This theory thus makes the empirically implausible predictions that the correlation in abundances in two plots of fixed area (a) does not decrease with increasing distance, and (b) does not asymptote to zero at large distances.

Within the context of maximum entropy theories, we note that both Banavar et al. (2010) and Haegeman and Etienne (2010) apply the maximum entropy principle in a manner that instead leads to a Poisson quadrat count distribution. Under these theories, the predicted spatial distribution of individuals is thus consistent with complete spatial randomness (see Table 1 and main text).

11 Additional Results

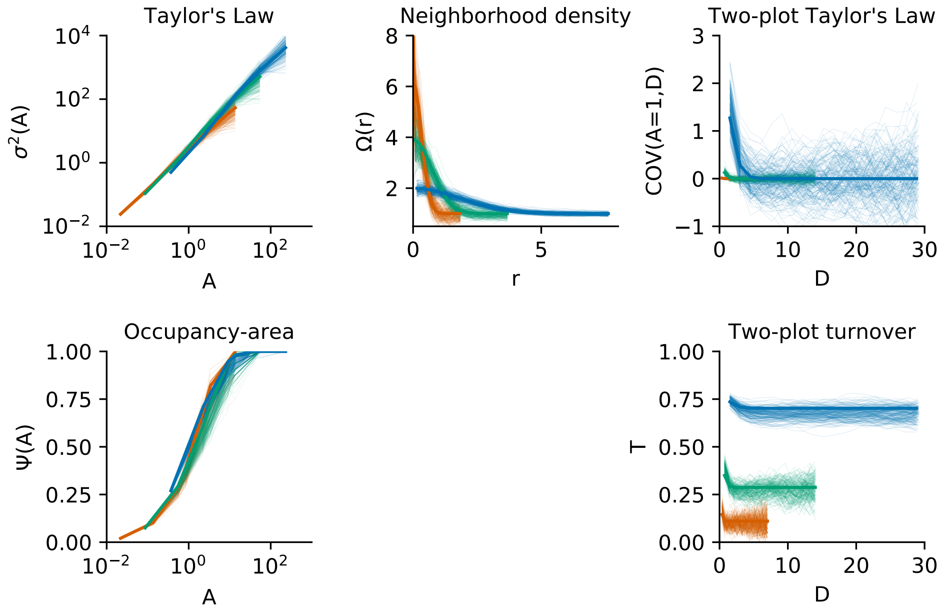

Figure SI1.1: Results of simulation study including all 600 simulated point pattern metrics Figure is analogous to Figure 4 in the main manuscript, except showing all 600 point pattern simulations (light colored lines) along with predictions (dark lines).

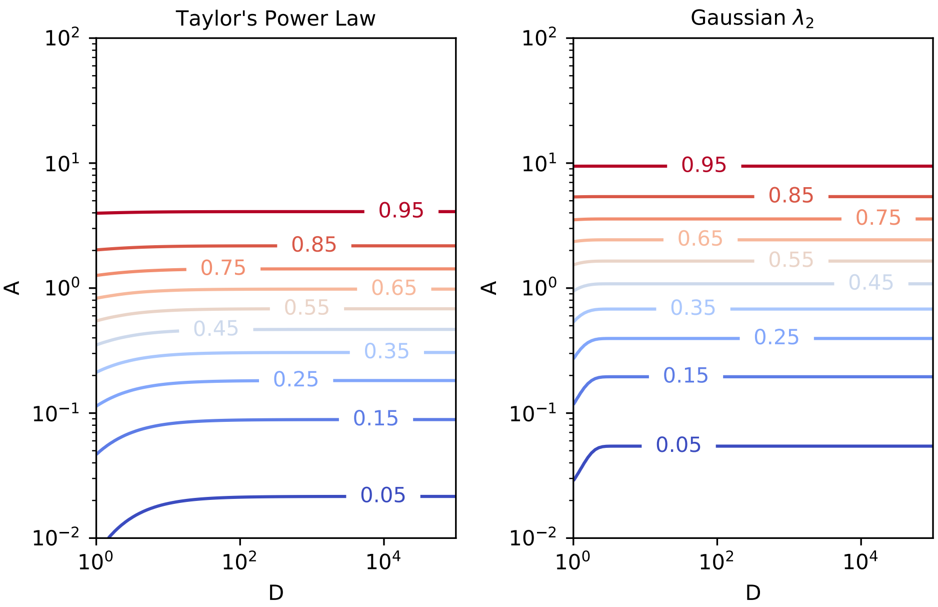

Figure SI1.2: Influence of plot area A and intraplot distance D on values of the two-plot turnover metric. Panels show turnover metric calculated for Taylor's power law in two-dimensions and a Gaussian second-order intensity. Plot area, as opposed to intraplot distance, can be seen to be a primary influence on values of the turnover metric in both examples. For a species with a power law Taylor's Law, for example, a plot of $A = 1$ has a turnover metric of 0.70 at $D = 1$, decaying to 0.66 at large D. This turnover at large D is approximately the same as the turnover at $D = 1$ for a plot of $A = 0.85$. For these parameters, a 15% difference in plot area, or equivalently expected species abundance, gives approximately the same change in the turnover metric as a several order of magnitude increase in interplot distance. Parameters are the same as those used in Figure 3 in the main text.

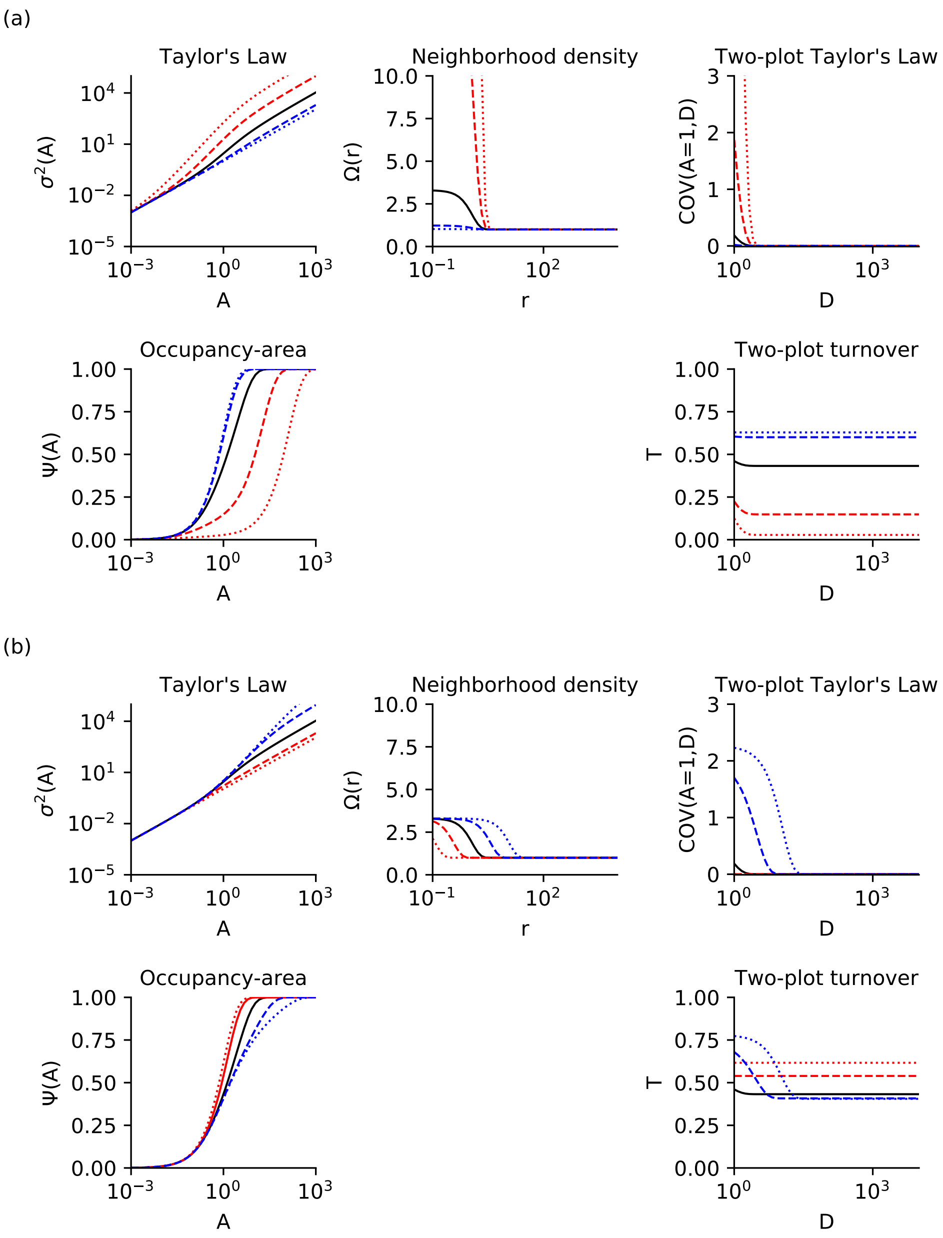

Figure SI1.3: Change in shape of metrics calculated from Gaussian second-order intensity with change in α and β parameters. Black lines in both subplots show predicted metrics for the case of a Gaussian-second order intensity function with parameters $\alpha = 2.7$ and $\beta = 0.8$, equal to the median parameters across the Barro Colorado Island data set. In (a), the parameter β is held constant at this value, and colored lines show predicted metrics for a one order of magnitude increase (red dashed), a two order of magnitude increase (red dotted), a one order of magnitude decrease (blue dashed), and two order of magnitude decrease (blue dotted) in the parameter α relative to the black line. In (b), similar calculations are performed but holding α constant and allowing β to vary. In general, large variation in β has less of an effect on the predicted metrics than variation in α. Figure 5 shows that disagreements in the estimate of β across empirical metrics are generally larger than disagreements in the estimate of α.

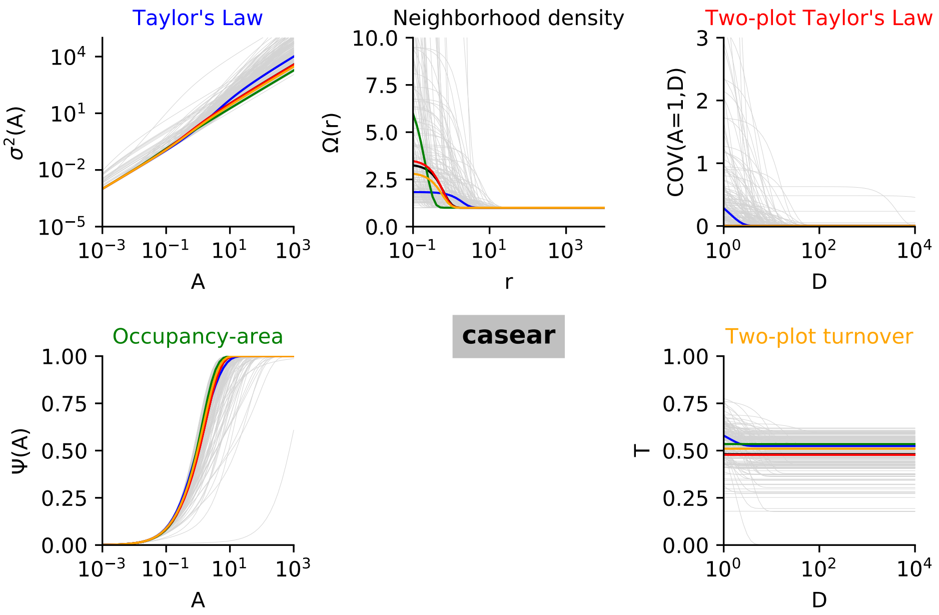

Figure SI1.4: Predicted shapes of five spatial metrics based on fits to empirical shapes of all five metrics, for species Casearia arborea. *Casearia arborea* has an abundance of 108 individuals in the Barro Colorado Island forest plot and is the species with median abundance in the lowest abundance tertile. Each panel shows five predictions for the named spatial metric, with each prediction based on parameters fitted from the empirical shape of one spatial metric (neighborhood density function: black, Taylor's Law: blue, occupancy area curve: green, two-plot Taylor's Law: red, turnover: orange). For example, in the Taylor's Law panel, the black line shows the predicted Taylor's Law based on fitted parameters from the empirical neighborhood density function, the green line shows the predicted Taylor's Law based on fitted parameters from the empirical occupancy area curve, etc. All fitting and predictions use the parametric forms of the five spatial metrics associated with the theoretical example of a Gaussian second-order intensity function (see main text). Overall, the five predictions for each metric can be seen to be relatively similar to each other compared to variation in best fitting metric shapes across all species (light gray lines). Similar plots for all 171 species analyzed in the Barro Colorado Island dataset are available in Supporting Information 4.

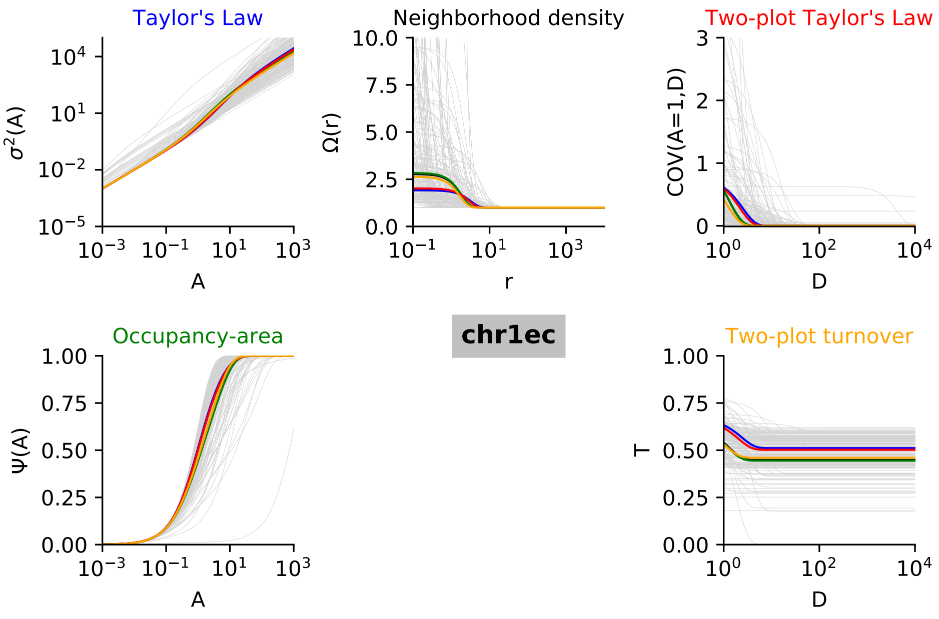

Figure SI1.5: Predicted shapes of five spatial metrics based on fits to empirical shapes of all five metrics, for species Chrysochlamys eclipes. *Chrysochlamys eclipes* has an abundance of 432 individuals in the Barro Colorado Island forest plot and is the species with median abundance in the middle abundance tertile. The interpretation of panels and lines in this figure is identical to that of Figure SI1.4. Overall, the five predictions for each metric can be seen to be relatively similar to each other compared to variation in best fitting metric shapes across all species. Predictions based on empirical fits to Taylor's Law and the two-plot Taylor's Law, however, can be seen to systematically deviate by a small amount from predictions based on fits to the other three metrics. This deviation is consistent with the overall patterns in Figure 5 in the main text, including both the low correlation between fitted parameters from Taylor's Law and several other metrics and the high correlation between fitted parameters from Taylor's Law and the two-plot Taylor's Law.

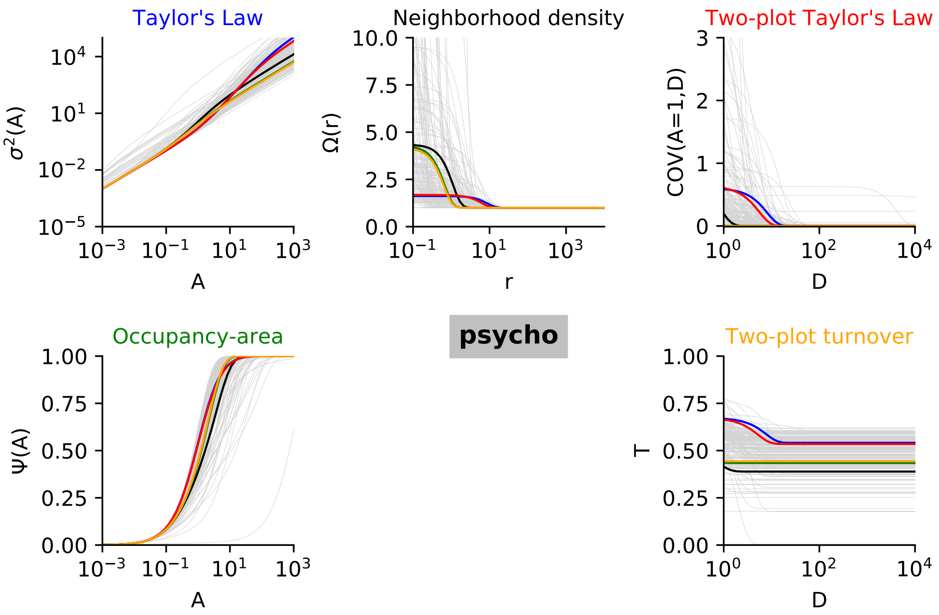

Figure SI1.6: Predicted shapes of five spatial metrics based on fits to empirical shapes of all five metrics, for species Psychotria horizontalis. *Psychotria horizontalis* has an abundance of 1,856 individuals in the Barro Colorado Island forest plot and is the species with median abundance in the highest abundance tertile. The interpretation of panels and lines in this figure is identical to that of Figure SI1.4. Similar to Figure SI1.4, predictions group into two sets, with those based on empirical fits to Taylor's Law and the two-plot Taylor's Law deviating from predictions based on fits to the other three metrics.

References

Banavar, J.R., Maritan, A. & Volkov, I. (2010). Applications of the principle of maximum entropy: From physics to ecology. *Journal of Physics: Condensed Matter*, 22, 063101.

Chu, D.P. (2006). Distance between random points in two rectangular cities. *Communications in Statistics - Simulation and Computation*, 35, 257–276.

Diggle, P.J. (2013). *Statistical Analysis of Spatial and Spatio-Temporal Point Patterns*. CRC Press.

Haegeman, B. & Etienne, R.S. (2010). Entropy maximization and the spatial distribution of species. *The American Naturalist*, 175, E74–E90.

Harte, J. (2011). *Maximum entropy and ecology: A theory of abundance, distribution, and energetics*. Oxford series in ecology and evolution. Oxford University Press, Oxford ; New York.

Harte, J. & Newman, E.A. (2014). Maximum information entropy: A foundation for ecological theory. *Trends in Ecology & Evolution*, 29, 384–389.

Harte, J., Zillio, T., Conlisk, E. & Smith, A.B. (2008). Maximum entropy and the state-variable approach to macroecology. *Ecology*, 89, 2700–2711.

He, F. & Gaston, K.J. (2003). Occupancy, spatial variance, and the abundance of species. *The American Naturalist*, 162, 366–375.

Illian, J., Penttinen, A., Stoyan, H. & Stoyan, D. (2008). *Statistical Analysis and Modelling of Spatial Point Patterns*. John Wiley & Sons, Ltd, Chichester, UK.

Jaynes, E.T. (1982). On the rationale of maximum-entropy methods. *Proceedings of the IEEE*, 70, 939–952.

Johnson, N.L., Kotz, S. & Balakrishnan, N. (1997). *Discrete Multivariate Distributions*. Wiley.

Mathai, A.M. (1999). *An Introduction to Geometrical Probability: Distributional Aspects with Applications*. Statistical Distributions & Models with Applications. CRC Press.

Mathai, A.M., Moschopoulos, P. & Pederzoli, G. (1999). Random points associated with rectangles. *Rendiconti del Circolo Matematico di Palermo*, 48, 163–190.

Weisstein, E.W. (2020). Square Line Picking. *Mathworld - A Wolfram Web Resource*.
