# Supplementary material for: A unified framework for species spatial patterns: Linking the occupancy area curve, Taylor's Law, the neighborhood density function and two‐plot species turnover
Source: Ecol Lett. 2021 Aug 4;24(10):2043–53. doi: 10.1111/ele.13788 (PMC8518128; doi:10.1111/ele.13788)
Supplement: Supplementary file 2 — Supplementary Material [file ELE-24-2043-s002.pdf]

A unified framework for species spatial patterns: Linking the occupancy area curve, Taylor's Law, the neighborhood density function, and two-plot species turnover

Supporting Information 2

Justin Kitzes[1], Micah Brush[2], Kyle Walters[1]

1 - Department of Biological Sciences, University of Pittsburgh
2 - Department of Physics, University of California, Berkeley

1 Approximating the second order intensity, $\lambda_2(r)$

In the 2D case, we will assume that we have a square plot with side length $h = \sqrt{A}$. We can use the square line picking distribution, the 2D extension of the line line picking distribution in Eq. 29 in Supporting Information 1, to write

$$E[R] = \int_0^h 2r\left(r^2 - 4rh + \pi h^2\right)\lambda_2(r)dr + \int_h^{\sqrt{2}h} 2r\left(4h\sqrt{r^2-h^2} - r^2 + (\pi-2)h^2 - 4h^2\arctan\left(\frac{\sqrt{r^2-h^2}}{h}\right)\right)\lambda_2(r)dr. \quad (1)$$

In 2D using a power law form of Taylor's Law ($\sigma^2 = a\mu^b$), we can also write

$$E[R] = \sigma^2 + \mu^2 - \mu = a\lambda^b h^{2b} + \lambda^2 h^4 - \lambda h^2 \quad (2)$$

where μ is the expected number of individuals in the plot, and λ is the average number of individuals per unit area. We can set the units of area such that $\lambda = 1$, but we will keep λ in the equations below for now.

Equating Eq. 1 and Eq. 2 gives us an expression for $\lambda_2(r)$, which we could solve in the corresponding 1D case. Here however, we cannot solve this equation exactly, and will proceed with an approximation to $\lambda_2(r)$.

In the square line picking distribution itself, the kernel in Eq. 1, the second term only contains about 2.5% of the probability weight. If we assume $\lambda_2(r)$ is decreasing with distance, then this second term will be very small. To approximate this equation, we will ignore the second term and instead extend the range of integration of the first integral to where the function intersects the x-axis, at a radius $k = \left(2-\sqrt{4-\pi}\right)h$. Equating this to Eq. 2 and rewriting in terms of k gives

$$\frac{a\lambda^b k^{2b}}{\left(2-\sqrt{4-\pi}\right)^{2b}} + \frac{\lambda^2 k^4}{\left(2-\sqrt{4-\pi}\right)^4} - \frac{\lambda k^2}{\left(2-\sqrt{4-\pi}\right)^2} = \int_0^k 2r\left(\frac{\pi k^2}{\left(2-\sqrt{4-\pi}\right)^2} - \frac{4kr}{2-\sqrt{4-\pi}} + r^2\right)\lambda_2(r)\,dr \quad (3)$$

To solve this, we take three derivatives with respect to k on both sides. This turns the above equation into the following differential equation

$$a(b-1)(2b-1)b\left(2-\sqrt{4-\pi}\right)^{2(1-b)}\lambda^b k^{2b-3} + \frac{6\lambda^2 k}{\left(2-\sqrt{4-\pi}\right)^2} = \left(\pi - 2\left(2-\sqrt{4-\pi}\right)\right)k^2\lambda_2'(k) + \left(3\pi - 4\left(2-\sqrt{4-\pi}\right)\right)k\lambda_2(k). \quad (4)$$

Solving this expression gives

$$\lambda_2(k) = \frac{ab(b-1)(2b-1)\lambda^b(2-\sqrt{4-\pi})^{2(1-b)}}{\pi(2b-1) - 4(b-1)(2-\sqrt{4-\pi})}k^{2(b-2)} + \frac{6\lambda^2}{(2-\sqrt{4-\pi})^2(2\pi-(2-\sqrt{4-\pi})^2)} + Ck^{-3+\frac{1}{1-\frac{\pi}{2(2-\sqrt{4-\pi})}}} \quad (5)$$

where C is the integration constant, which we set to 0 to ensure that the integral in Eq. 3 converges. This requirement also enforces that $b \geq 1$.

This expression looks quite complicated, but importantly the only term with k dependence is the first term. This means that we can write

$$\lambda_2(r) \approx cr^{2(b-2)} + \lambda^2 \tag{6}$$

where c is a numerical constant that depends on a and b. Here we have also set the coefficient in front of the constant term to be exactly 1, rather than its numerical value of 1.015 from Eq. 5, so that $\lim_{r\to\infty} \lambda_2(r) = \lambda^2$, which is the correct large r behavior for the second order intensity function. This also ensures that the covariance calculated with this $\lambda_2(r)$ has the correct limit when the plots are very far apart. This equation is a natural extension to $\lambda_2(r)$ in 1D, since the r dependence changes to r^2.

This expression does not recover the quadratic term on the left hand side of Eq. 4 as we took three derivatives before solving for $\lambda_2(r)$. This is as in the 1D case with the linear term. Here, by inspection, we should add

$$-\frac{\lambda \delta(r)}{\pi r} \tag{7}$$

to $\lambda_2(r)$.

With this approximation of $\lambda_2(r)$, we obtain the following expression for σ^2

$$\sigma^2 = \int_0^h 2r\left(r^2 - 4rh + \pi h^2\right) cr^{2(b-2)} dr + \int_h^{\sqrt{2}h} 2r\left(4h\sqrt{r^2-h^2} - r^2 + (\pi-2)h^2 - 4h^2 \arctan\left(\frac{\sqrt{r^2-h^2}}{h}\right)\right) cr^{2(b-2)} dr. \tag{8}$$

We plot this equation compared to σ^2 directly from Taylor's Law in Fig. 1. The error in this approximation for b close to 2 is around 1% and is subpercent for smaller b.

2 Approximating the covariance $\mathrm{C}(A,D)$ in 2D

It isn't obvious how to extend Eq. 1 to the two plot case, so instead we will use a different formulation. Rather than the square line picking distribution, consider two vertical lines a distance d apart and integrate over all distances on those two lines, and then integrate over all horizontal distances between the two lines. This is as explained in Supporting Information 1, and leads to

$$E[R] = \int_0^h 2(h-d) \int_d^{\sqrt{h^2+d^2}} 2r\left(\frac{h}{\sqrt{r^2-d^2}} - 1\right) \lambda_2(r)\, dr\, dd, \tag{9}$$

which is equivalent to Eq. 1 with a change of integration order. However this expression is easier to extend to 2D by changing the bounds of integration as in the 1D case.

First, we assume that the two plots are on the same axis and separated by a distance D. The integral over r stays the same as in the single plot case, as it is just the integral over the distances on the vertical line segments. The integral over d is the same as the integral over the line line picking distribution in the 1D case, so we can extend it in the same way. Rather than a single integral over $2(h-d)$, we get two integrals, $\int_D^{D+h} (d-D) I_r(d)\, dd$ and $\int_{D+h}^{D+2h} (D+2h-d) I_r(d)\, dd$, where $I_r(d) = \int_d^{\sqrt{h^2+d^2}} 2r\left(\frac{h}{\sqrt{r^2-d^2}} - 1\right) \lambda_2(r)\, dr$.

The two plot equation is then

$$\mathrm{C}(A,D) + \lambda^2 h^4 = \int_D^{D+h} (d-D) \int_d^{\sqrt{h^2+d^2}} 2r\left(\frac{h}{\sqrt{r^2-d^2}} - 1\right) \lambda_2(r)\, dr\, dd + \int_{D+h}^{D+2h} (D+2h-d) \int_d^{\sqrt{h^2+d^2}} 2r\left(\frac{h}{\sqrt{r^2-d^2}} - 1\right) \lambda_2(r)\, dr\, dd. \tag{10}$$

We now substitute in for $\lambda_2(r)$ assuming the functional form in Eq. 6. We will use $\lambda_2(r) = cr^{-\gamma} + \lambda^2$, where $\gamma = 2(2-b)$. Integrating the constant term on the right hand side gives $\lambda^2 h^4$, which cancels with the corresponding term on the left hand side. This ensures that $\lim_{D\to\infty} \mathrm{C}(A,D) = 0$.

For the power law term, the integral over r gives

$$I_r(d) = \frac{c}{1-\gamma/2}\left(d^{2-\gamma} - (d^2+h^2)^{1-\gamma/2}\right) + \frac{2ch^2}{(d^2+h^2)^{\gamma/2}}\,{}_2F_1\left(\frac{\gamma}{2}, 1; \frac{3}{2}; \frac{h^2}{d^2+h^2}\right), \tag{11}$$

where ${}_2F_1$ is the Hypergeometric function. We then need to solve the integral over d, which we can do analytically in a few special cases, however these cases are specific and still give complicated solutions.

More generally we can approximate $I_r(d)$ in the case $D >> h$ and then do the integral over d. This corresponds to the case that the distance between plots is much larger than the plots themselves. In this limit, we get

$$I_r(d) \to cd^{-\gamma}h^2\left(1 - \frac{\gamma}{12}\left(\frac{h}{d}\right)^2 + \frac{\gamma(\gamma+2)}{120}\left(\frac{h}{d}\right)^4 + \dots\right). \tag{12}$$

This approximation makes sense in that the first order term would be correct if the plots were single points. Keeping only the next highest order term gives the following integral

$$\mathrm{C}(A,D) = ch^2\int_D^{D+h}(d-D)d^{-\gamma}\left(1-\frac{\gamma}{12}\left(\frac{h}{d}\right)^2\right)dd + ch^2\int_{D+h}^{D+2h}(D+2h-d)d^{-\gamma}\left(1-\frac{\gamma}{12}\left(\frac{h}{d}\right)^2\right)dd,$$

which can be solved analytically and gives

$$\mathrm{C}(A,D) = ch^2\left(\frac{1}{(\gamma-1)(\gamma-2)}\left(D^{2-\gamma} - 2(D+h)^{2-\gamma} + (D+2h)^{2-\gamma}\right) - \frac{h^2}{12(\gamma+1)}\left(D^{-\gamma} - 2(D+h)^{-\gamma} + (D+2h)^{-\gamma}\right)\right). \tag{13}$$

Fig. 2 shows the results of approximating $I_r(d)$ for various a and b. The error in this approximation is at the subpercent level for $D \geq h$.

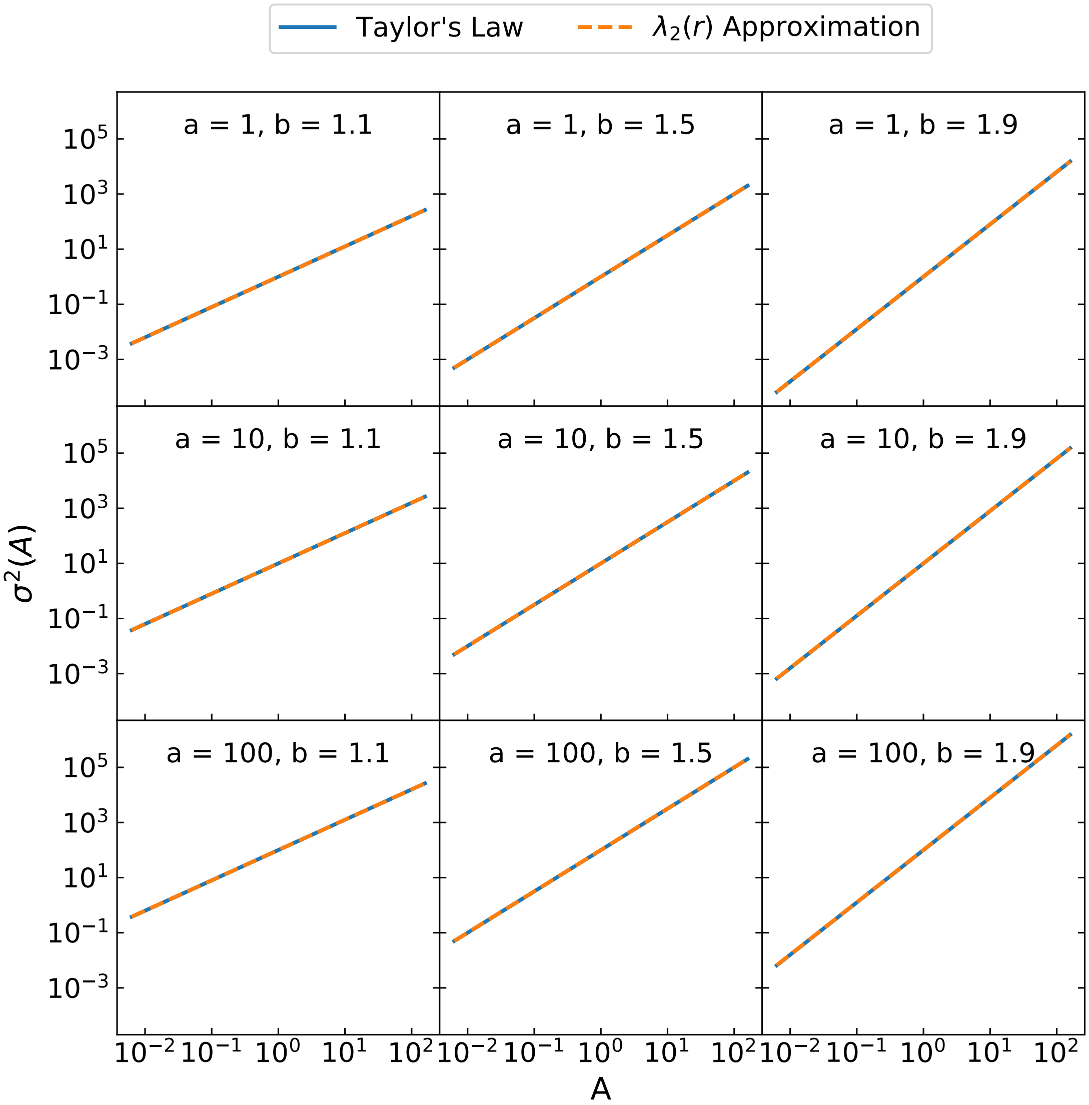

Figure 1: σ^2 calculated with a power law form Taylor's Law ($\sigma^2 = a\mu^2$) and with the approximation for $\lambda_2(r)$ (Eq. 8) for various values of a and b.

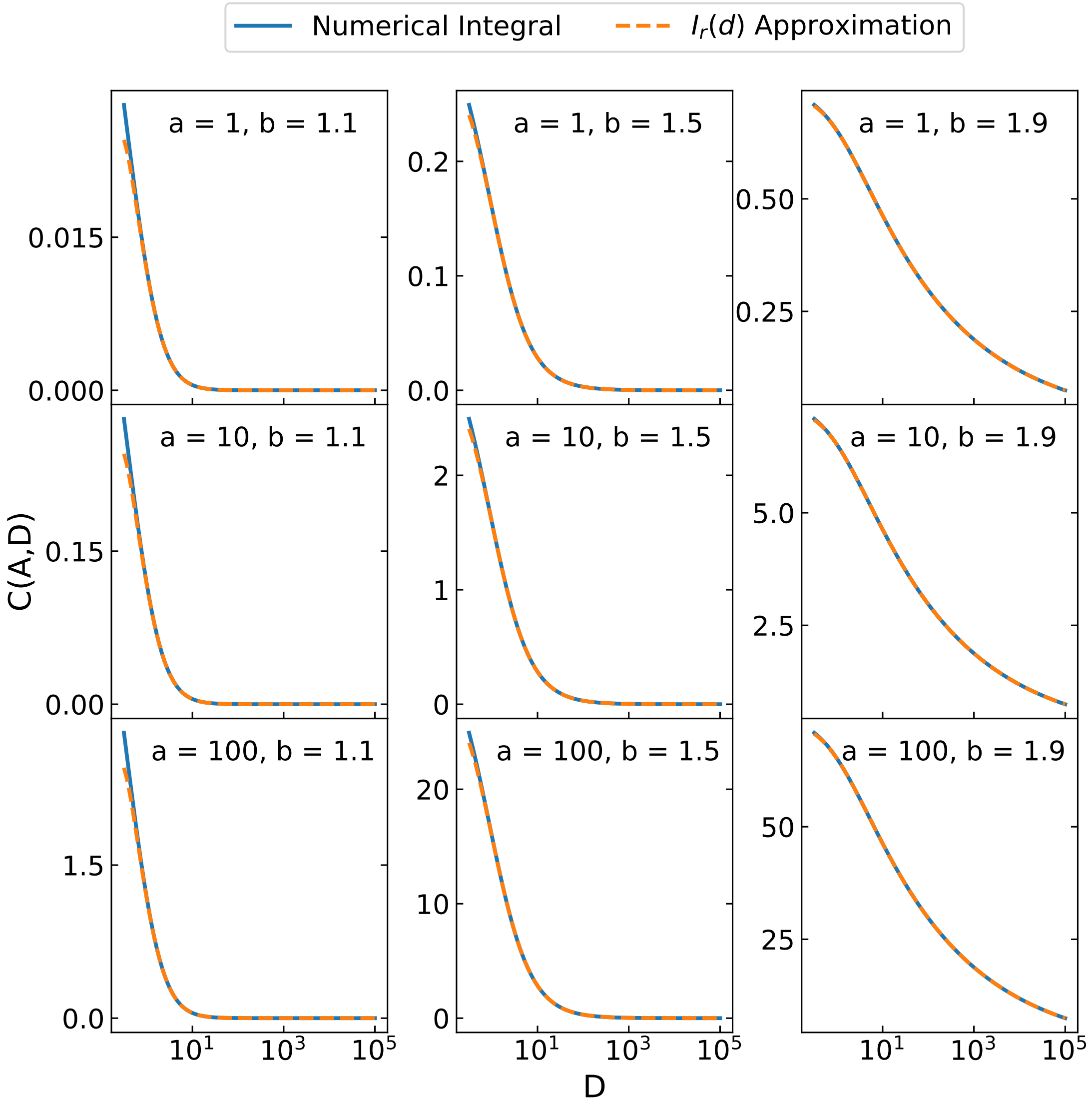

Figure 2: Numerical integration of Eq. 11 compared to the result from the first order approximation in Eq. 13.
