# Supplementary material for: A unified framework for species spatial patterns: Linking the occupancy area curve, Taylor's Law, the neighborhood density function and two‐plot species turnover
Source: Ecol Lett. 2021 Aug 4;24(10):2043–53. doi: 10.1111/ele.13788 (PMC8518128; doi:10.1111/ele.13788)
Supplement: Supplementary file 3 — Supplementary Material [file ELE-24-2043-s001.pdf]

A unified framework for species spatial patterns: Linking the occupancy area curve, Taylor's Law, the neighborhood density function, and two-plot species turnover

Supporting Information 3

Justin Kitzes[1], Micah Brush[2], Kyle Walters[1]

1 - Department of Biological Sciences, University of Pittsburgh
2 - Department of Physics, University of California, Berkeley

Introduction

This document provides a detailed description of the methods and an overview of the code used to conduct the simulation study and empirical analysis presented in the main text. The code to execute these analyses can be found in a ZIP file as Supporting Information 4. The code is contained mainly in a series of Jupyter notebooks that are written mostly in Python 3 (notebooks using R kernels are specifically noted below).

Supporting Information 4 consists of a main directory that contains six subdirectories that should be viewed and/or executed in numerical order. This main directory also contains a file `predictfns.py`, which contains functions that calculate the predicted forms of the five spatial metrics for the theoretical examples of a power law Taylor's Law and Gaussian second-order intensity. Equations for these metrics are given in Table 1 and the main text. This file is imported by several notebooks.

1-plot-data

The first directory analyzes data from the 2015 census of the Barro Colorado Island (BCI) tropical forest plot. The outputs from this directory are gridded, plot-based abundance counts for each species, the α and β parameters of the best fitting Gaussian second-order intensity function for each species, and calculations of the median α and β parameters for each abundance tertile in this data (which are used subsequently for the simulation study). The file `bci.tree8.rdata` is taken from the current version of the BCI census data found on Dryad (https://doi.org/10.15146/5xcp-0d46) and forms the basis of the empirical analysis.

The notebook `generate-grid-data` uses an R kernel to analyze the BCI data using the package `spatstat`. Data from the BCI census for 2015 are loaded and subsetted to only living trees, with trees occupying the same spatial coordinate removed, leaving 221,523 individuals. For each species with over 70 individuals, the dimensions of the plot, length units, and coordinate locations are rescaled such that $\lambda = 1$ (see Supporting Information 1). An empirical pair correlation function, equivalent to the second-order intensity in these scaled units, is then calculated for each species. The best fitting α and β parameters for each species are calculated using the *thomas.pcf* function. The entire landscape is then gridded into square cells of varying resolutions (side lengths in original units of meters: 10, 25, 50, 62.5, 100, 125, 250), and matrices with the abundance of each species in each cell are saved. These gridded data form the basis for analysis of the four plot-based spatial metrics (all except the neighborhood density function).

The notebook `visualize-fits` uses the fitted α and β parameters saved by `generate-grid-data` to determine the median abundance n, α, and β for each of three abundance tertiles in the BCI data. After exploratory plotting, these median values are calculated and printed. This notebook also creates and saves a plot of the empirical and best fitting Gaussian second-order intensity function for each species.

2-sim-thomas

The second directory simulates point patterns from a Thomas process model for use in the simulation study. The outputs from this directory are similar to those from the first directory, but calculated instead from the simulated point patterns: gridded abundance counts for each simulation, best fitting α and β parameters from the second-order intensity function, and plots of the measured and best fitting second-order intensity function.

The notebook `thomas-simulation` uses an R kernel to simulate 200 replicate point patterns for each of three idealized species, which have α and β parameters drawn from the medians of the BCI abundance tertiles. The abundance, α,

and β parameters for each species are declared along with the raw dimensions of the landscape (the same as the BCI tropical forest plot). All areas, lengths, and coordinates are once again rescaled such that $\lambda = 1$. The parameters n, α, and β are converted into the usual parameters of the Thomas process, which are scale, kappa, and mu. For each idealized species, 200 replicate point patterns are created using a rejection method that only accepts as valid a point pattern with a simulated abundance that is within 2% of the target abundance. For valid point patterns, the empirical pair correlation function (equivalent to the second-order intensity in scaled units) is then calculated and the best fitting α and β parameters for the Thomas process are fitted using *thomas.pcf*. Finally, using the same approach as in the first directory, gridded abundance data for each simulated point pattern are calculated and saved.

The notebook `visualize-fits` is structured similarly to the notebook with the same name in the first directory. This notebook explores the best fitting α and β parameters for each simulation, calculates the median values of these parameters for each idealized species, and makes and saves a plot for each simulated point pattern showing the measured and best fitting second-order intensity functions. These results show that due to stochasticity, the median fitted parameters of the 200 simulations do not necessarily match the target parameters for that idealized species. In the main manuscript, we report small but non-negligible bias in the median parameters of the simulations as compared to the target parameters for two of the three idealized species.

3-single-plot-metrics

The third directory calculates the single plot metrics (Taylor's Law and occupancy area curve) across plot areas for all BCI species and Thomas process simulations. The outputs from this directory are CSV files, one for each species or simulation, reporting the shape of these two metrics as calculated from the previously saved gridded abundance data.

The notebook `calc-single-metrics` first loads abundance data for both the BCI species and simulations and concatenates these tables. It then loops through each row and each subplot area to calculate the variance of abundance in all grid cells and fraction of grid cells occupied. The subplots are square plots with side lengths (in original untransformed meters) of 10, 25, 50, 62.5, 100, 125, and 250 m.

4-two-plot-metrics

The fourth directory calculates the two plot metrics (two-plot Taylor's Law and turnover) for a single, fixed plot area across interplot distances for all BCI species and Thomas process simultaions. The outputs from this directory are CSV files, one for each species or simulation, reporting the shape of these two metrics as calculated from the previously saved gridded abundance data.

The notebook `calc-two-metrics` first loads abundance data for both the BCI species and simulations and concatenates these tables. It then declares a single subplot area (raw side length 25 m) to use as the fixed area, so that the two plot metrics can be calculated as a function of distance (see Figure 6 and the main manuscript for a discussion of the relative importance of plot area and interplot distance). The notebook next loops through each row and each possible interplot distance to calculate the covariance in abundance and the turnover metric. Distances are whole number multiples of plot side length, and pairs of plots are always located with one side lying on the same axis to match the assumptions of the theoretical examples.

5-fit-alpha-beta

The fifth directory finds the best fitting α and β parameters for the four spatial metrics other than the second-order intensity (for which best fitting parameters were already found in notebooks in the first and second directories), assuming that these metrics follow the equations for a species exhibiting a Gaussian second-order intensity. The output from this directory is a table of the best fitting parameters calculated from each spatial metric for each species or simulation.

The notebook `fig-alpha-beta` loads abundance and fitted values of α and β from the second-order intensity function. It then loops through each species or simulation and finds the best fitting α and β parameters for each of the other four spatial metrics by minimizing the sum of squares between the empirical metric and the theoretical form of that metric for a species with a Gaussian second-order intensity. Fitting is done using the `curve_fit` function in `scipy.optimize`. This function implements bounded optimization, and we note that in several cases, it appears that the fit between the empirical and predicted metrics was poor enough that the best fitting parameters were near the upper end of the bounded range, which was set to 10,000. We chose not to remove these species from further analysis, but instead recognize that

the Spearman's correlation coefficient for the empirical analysis in particular minmizes the influence of these outliers by considering only the rank of parameter values.

6-figures

The sixth directory creates Figures 3-5 from the main text and Figures SI1.1-SI1.6 from Supporting Information 1. The output from this directory are these figures, saved in PDF format. Note that in `fig-thomas`, various lines in the code can be commented to plot the predicted values of each metric, the median values of the simulated metrics, or the values of the metrics for each simulation. The subdirectory `spp-preds` contains the equivalent to Figure SI1.4 for all 171 species in the Barro Colorado Island data set.
