# Supplementary material for: A unified framework for species spatial patterns: Linking the occupancy area curve, Taylor's Law, the neighborhood density function and two‐plot species turnover
Source: Ecol Lett. 2021 Aug 4;24(10):2043–53. doi: 10.1111/ele.13788 (PMC8518128; doi:10.1111/ele.13788)
Supplement: Supplementary file 4 — Supplementary Material [file ELE-24-2043-s004.zip › calcs/6-figures/bci-spearman.pdf]

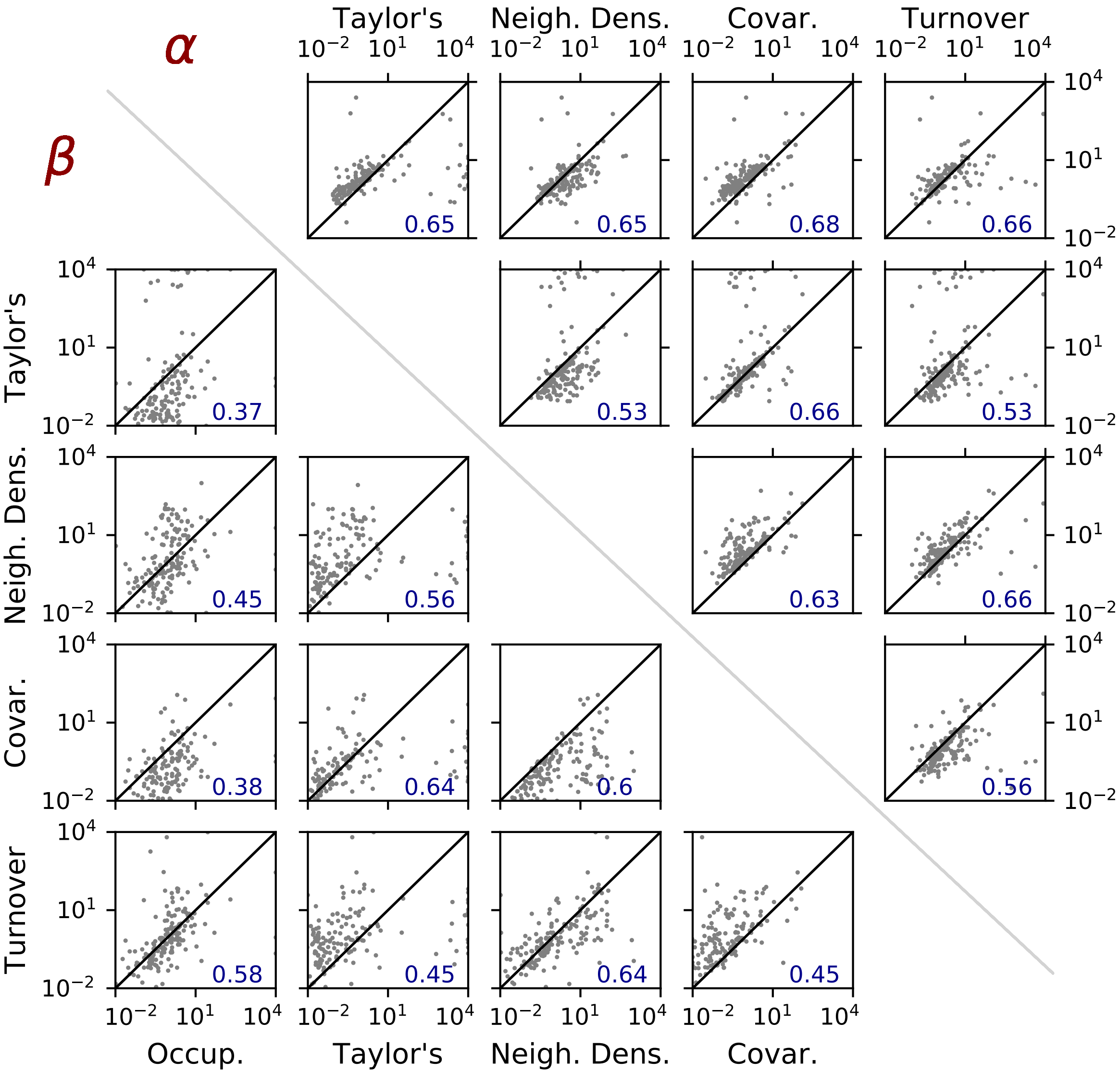

α
β
Taylor's
Neigh. Dens.
Covar.
Turnover
Occup.
0.65
0.65
0.68
0.66
0.37
0.53
0.66
0.53
0.45
0.56
0.63
0.66
0.38
0.64
0.6
0.56
0.58
0.45
0.64
0.45
10^{-2}
10^{1}
10^{4}
