# Supplementary figures and images for: A unified framework for species spatial patterns: Linking the occupancy area curve, Taylor's Law, the neighborhood density function and two‐plot species turnover
Source: Ecol Lett. 2021 Aug 4;24(10):2043–53. doi: 10.1111/ele.13788 (PMC8518128; doi:10.1111/ele.13788)

Taylor's Law

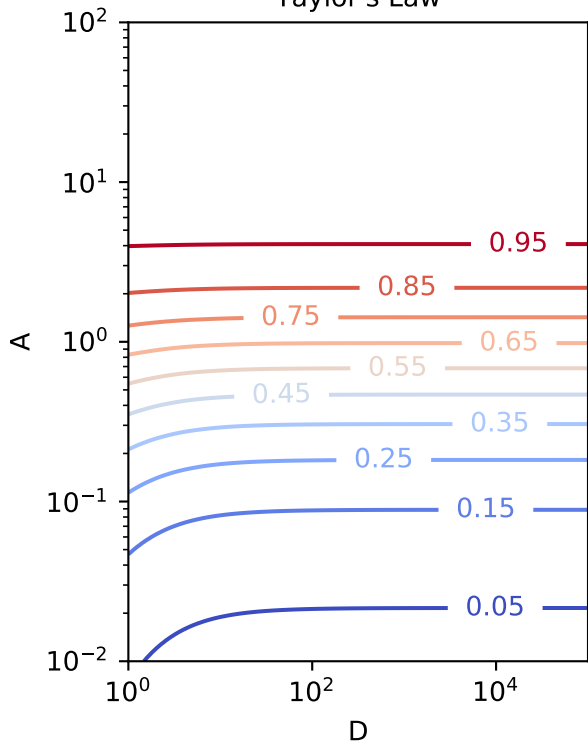Gaussian  $\lambda_2$ 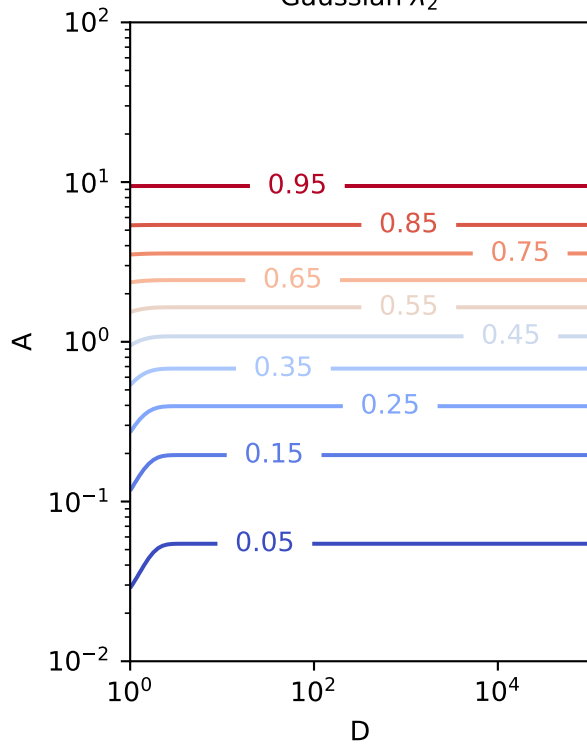

Supplement: Supplementary file 4 — Supplementary Material [file ELE-24-2043-s004.zip › calcs/6-figures/AD.pdf]

(a)

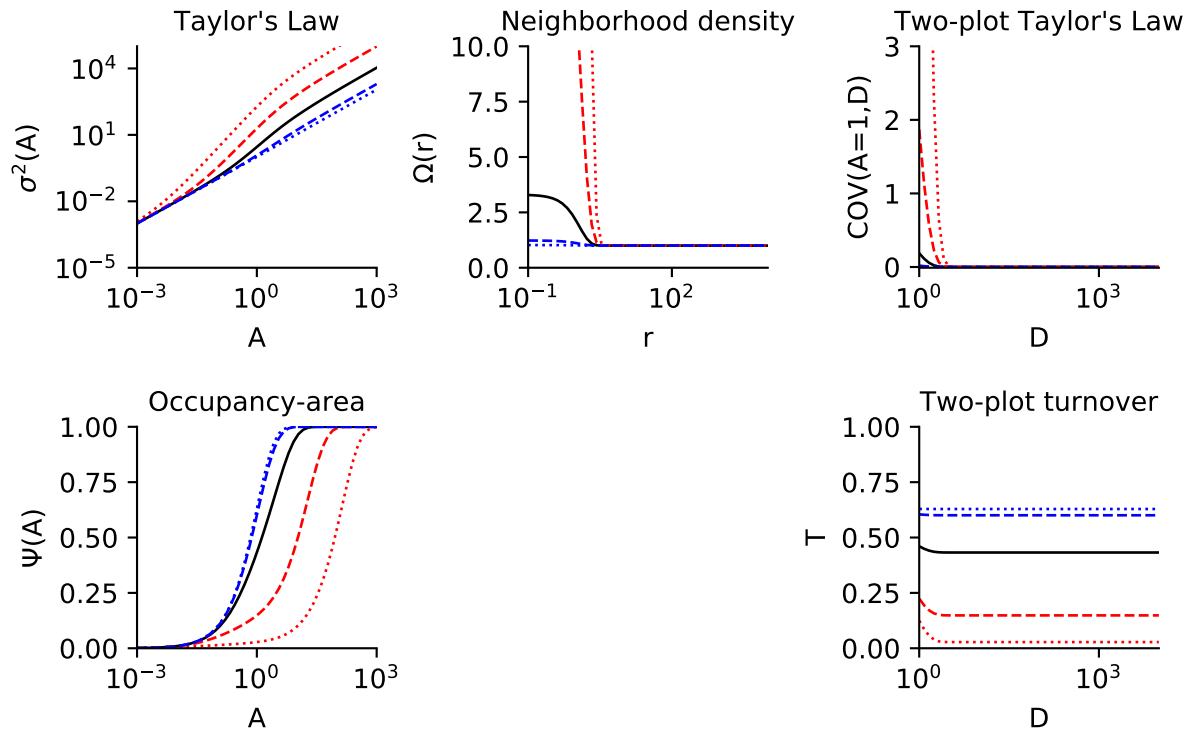

Supplement: Supplementary file 4 — Supplementary Material [file ELE-24-2043-s004.zip › calcs/6-figures/alpha_var.pdf]

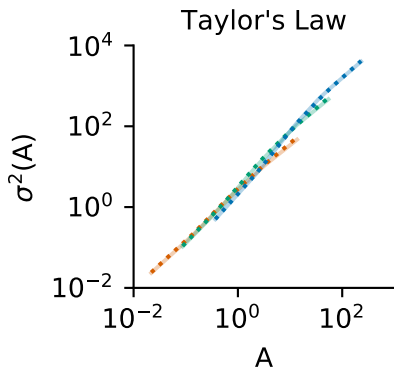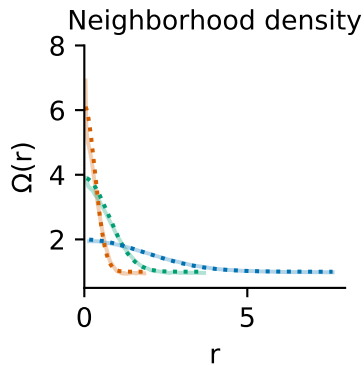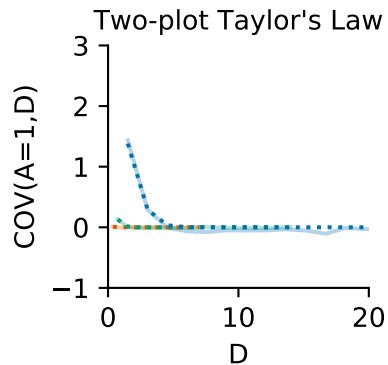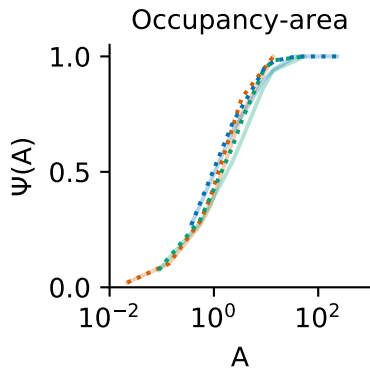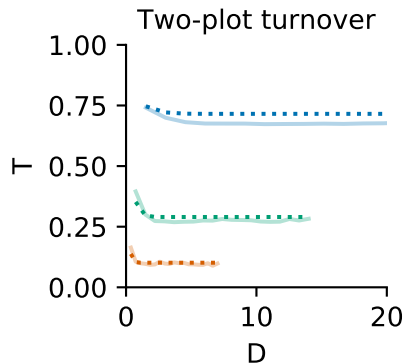

Supplement: Supplementary file 4 — Supplementary Material [file ELE-24-2043-s004.zip › calcs/6-figures/thomas-sim-vs-pred.pdf]

Taylor's Law

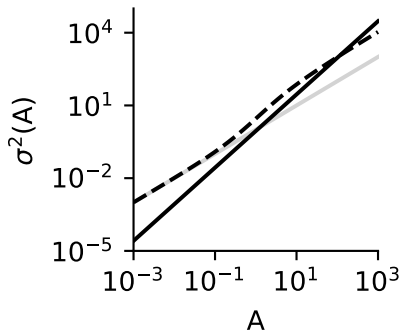

Neighborhood density

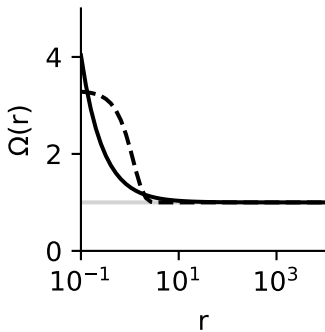

Two-plot Taylor's Law

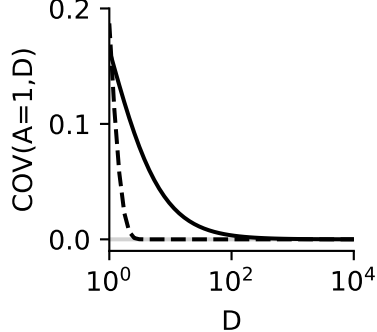

Occupancy-area

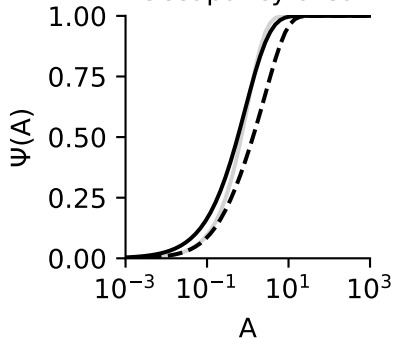

Two-plot turnover

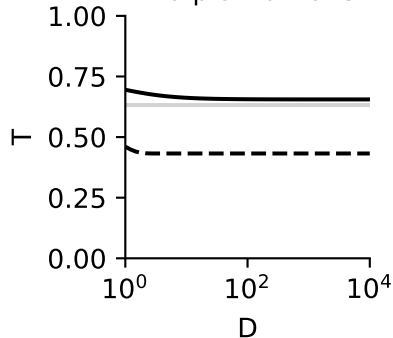

Supplement: Supplementary file 4 — Supplementary Material [file ELE-24-2043-s004.zip › calcs/6-figures/theory.pdf]

(b)

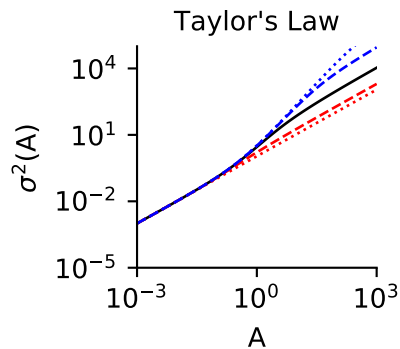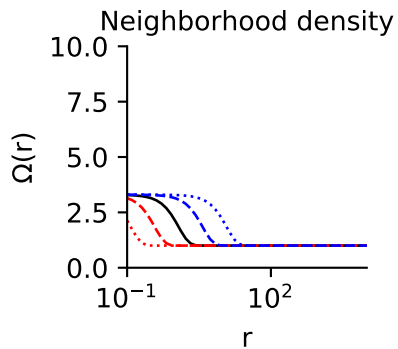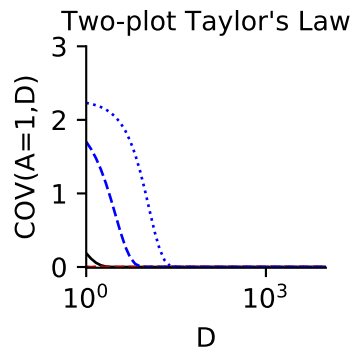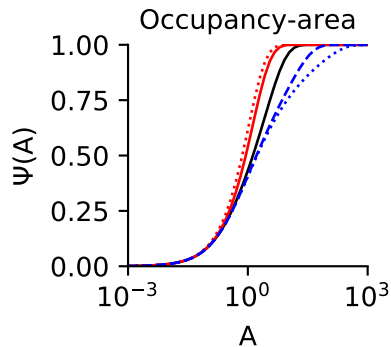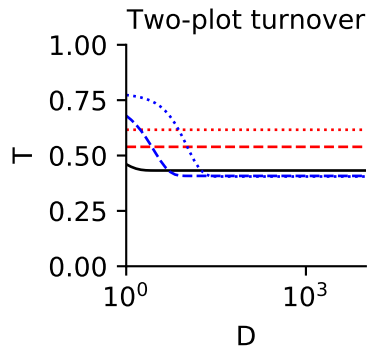

Supplement: Supplementary file 4 — Supplementary Material [file ELE-24-2043-s004.zip › calcs/6-figures/beta_var.pdf]

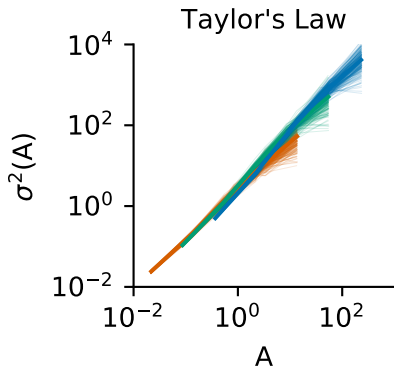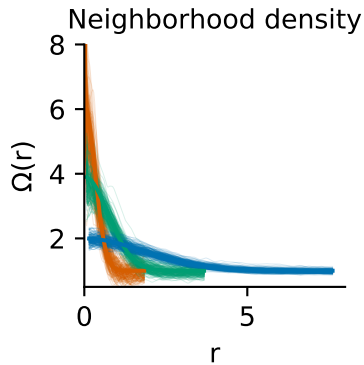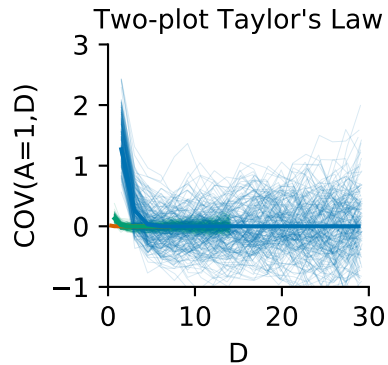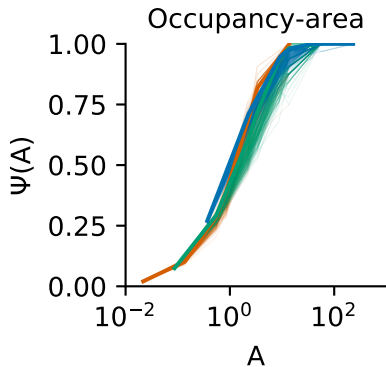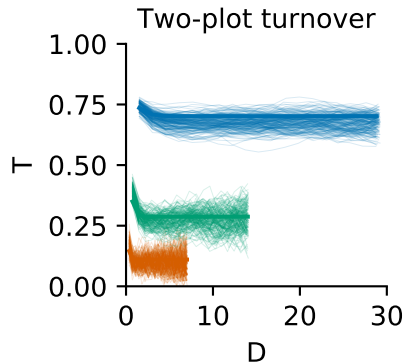

Supplement: Supplementary file 4 — Supplementary Material [file ELE-24-2043-s004.zip › calcs/6-figures/thomas-sim-vs-pred-all.pdf]
